# Chemical and Biological Determinants of PEG-Lipid Shedding From Therapeutic Nanoparticles

**DOI:** 10.1002/smll.75613

**Published:** 2026-09-10

**Authors:** Ethan P. Cisneros, Aadya S. Wijesekera, Lisa R. Volpatti

**Affiliations:** 1 Department of Biomedical Engineering, Northwestern University, Evanston, Illinois, USA; 2 Department of Chemical and Biological Engineering, Northwestern University, Evanston, Illinois, USA; 3 Biohub, Chicago, Illinois, USA; 4 International Institute for Nanotechnology, Northwestern University, Evanston, Illinois, USA

**Keywords:** drug delivery, formulation, immunoengineering, nanoparticle, PEG-lipids

## Abstract

Polyethylene glycol (PEG)-lipids are widely used to engineer the interfacial properties of lipid-based nanomaterials, where they play a central role in governing colloidal stability and biological interactions. By forming a steric barrier at the nanoparticle surface, PEG-lipids reduce protein adsorption and extend circulation times. Additionally, PEG-lipids are increasingly functionalized with targeting moieties, representing a primary strategy to enable selective delivery. However, accumulating evidence indicates that PEG-lipids are not static components but instead undergo dynamic dissociation, or “shedding,” upon exposure to biological environments. This interfacial exchange process alters nanoparticle behavior, promoting protein adsorption and accelerating clearance, while also enabling cellular interactions that facilitate uptake. Here, we present a conceptual framework for understanding PEG-lipid shedding as a coupled thermodynamic and kinetic process influenced by lipid chemical structure, nanocarrier membrane properties, and interactions with biomolecules. By synthesizing recent findings across experimental systems, we identify key factors that link PEG-lipid composition and nanoparticle architecture to desorption kinetics and biological outcomes. These insights provide guidance for the design of PEGylated nanomaterials with improved control over interfacial stability, immune recognition, and functional performance.

## 1 | Background: The Problem with PEG-Lipid Shedding

Lipid-based nanoparticles provide a versatile platform for delivering therapeutics that face challenges in stability, solubility, or cellular uptake [1]. Their chemical diversity allows them to form distinct structures capable of encapsulating complex cargo. Liposomes, for example, are self-assembled vesicles with a phospholipid-cholesterol bilayer that can carry both hydrophilic and hydrophobic molecules [2, 3]. Clinically approved formulations, such as doxorubicin liposomes (Doxil), improve the pharmacokinetics and biodistribution of small molecule therapeutics in certain cancers and infections [2–4].

In contrast to vesicular liposomes, lipid nanoparticles (LNPs) form smaller, more compact structures. LNPs can include ionizable or cationic lipids that form inverse lipid micelles internally, strongly complexing nucleic acid cargo [2]. These particles protect nucleic acids from degradation by extracellular ribonucleases and enable efficient intracellular delivery [5]. Compared with viral vectors, LNPs are less immunogenic, making them attractive for therapeutic nucleic acid delivery [6].

A key component of lipid-based nanoparticles is the PEG-lipid, which provides colloidal stability [7]. PEG-lipids consist of a hydrophilic polyethylene glycol (PEG) head group and a hydrophobic acyl chain tail that anchors them into the nanoparticle surface through hydrophobic interactions [8]. In addition to providing stability, the “stealth” PEG coating reduces protein adsorption and formation of the protein corona and lowers uptake by the mononuclear phagocyte system, which is responsible for the clearance of foreign particles [5].

Once in contact with biological fluids, PEG-lipids can undergo “shedding,” a process in which they detach from the nanoparticle surface [9]. PEG-lipid shedding is influenced by both formulation-dependent properties that determine lipid retention within the nanoparticle and environmental factors that promote transfer to lipoproteins, cell membranes, and other lipid-containing assemblies. This shedding can compromise nanoparticle stability, reduce delivery efficiency, cause off-target effects, and lead to premature clearance [10, 11]. On the other hand, PEG-lipid shedding enables nanoparticle interaction with target cells, facilitating uptake and functional delivery [12]. For example, Onpattro, the first US FDA-approved RNA interference therapy using LNP technology, relies on PEG-lipid desorption and subsequent adsorption of apolipoprotein E (ApoE) for hepatocyte-specific delivery [13]. ApoE is a lipid-binding protein involved in lipid transport and metabolism that acts as an endogenous targeting ligand, directing LNPs to the liver. While PEG is initially required for nanoparticle stability, Onpattro uses a rapidly desorbing PEG-lipid that dissociates after injection, allowing ApoE adsorption onto the nanoparticle surface [13]. More generally, PEG-lipid shedding is a delicate balancing act as premature shedding enhances clearance mechanisms while delayed shedding blocks cellular uptake.

Following the clinical approval of Onpattro, two PEGylated LNP-based mRNA vaccines were approved by the FDA for prevention of SARS-CoV-2 infection. Both formulations (BNT162b2 and mRNA-1273) utilize PEG-lipids for stability, alongside ionizable lipids, cholesterol, and phospholipid components for intramuscular delivery. The clinical success of these RNA therapies has catalyzed extensive research aimed at extending systemic LNP delivery beyond the liver to extrahepatic tissues, including through engineering of targeted LNPs with modified surface chemistries [14].

LNPs have been engineered to enable targeting through passive, endogenous, and active mechanisms [15–17]. Passive targeting exploits nanoparticle physical properties, such as size and shape, to direct biodistribution and cellular uptake [18]. For example, smaller nanoparticles (<50 nm) are thought to penetrate tumors more effectively through the enhanced permeability and retention effect [19–21]. Endogenous targeting similarly leverages physicochemical properties by modulating the protein corona to achieve organ and cell specificity [15, 18]. Selective organ targeting (SORT) LNPs exemplify this approach by incorporating an additional permanent cationic or anionic lipid to achieve tissue-specific tropism. For example, anionic SORT lipids have been associated with increased splenic targeting via recruitment of *β*2-glycoprotein I [22, 23].

Active targeting strategies incorporate targeting ligands, such as antibodies or peptides [24–28], most commonly by conjugating them to chemically functionalized PEG-lipids [29]. For example, Billingsley et al. developed CD3- and CD7-targeted LNPs that achieved significantly higher accumulation in the spleen versus liver [28]. However, these ligand-bearing PEG-lipids are noncovalently associated with the LNP and may readily desorb, reducing targeting efficiency (Figure 1). Strategies to mitigate shedding include using PEG-lipids with higher affinity for the nanoparticle or increasing PEG-lipid content to extend circulation time, enhancing systemic exposure and improving targeting [30].

Although chemical properties of PEG-lipids, such as lipid tail length, are known to influence apparent PEG-lipid retention and desorption behavior in vivo, the biological mechanisms governing this process remain poorly understood. In this review, we examine how PEG-lipid chemistry and biological factors may influence shedding, including the PEG head group, head-tail linkage, and lipid portion, as well as potential contributions from the protein corona, anti-PEG antibodies, and the accelerated blood clearance phenomenon. Understanding these factors is critical for developing targeted extrahepatic therapeutics for immunotherapies and other disease areas.

## 2 | Effects of PEG-Lipid Chemistry and Nanoparticle Formulation on Shedding

### 2.1 | Lipid Tail

Because PEG-lipids are anchored within the nanoparticle bilayer via hydrophobic interactions, variation in lipid tail chemistry is a primary determinant of PEG-lipid shedding and downstream biological performance. Here, we review how acyl length, branching, and degree of saturation influence these processes in lipid-based nanoparticles.

#### 2.1.1 | Acyl Length

Acyl chain length is the most widely studied determinant of PEG-lipid shedding. Longer acyl chains are generally associated with reduced PEG-lipid shedding in circulation due to stronger hydrophobic anchoring within the lipid bilayer, which limits exchange with serum proteins [9]. Conversely, shorter acyl chain lengths are more readily displaced, leading to enhanced shedding [9]. This length-dependent desorption underlies key tradeoffs in nanoparticle pharmacokinetics, biodistribution, and functional delivery.

Quantitative pharmacokinetic analyses illustrate the magnitude of this effect. Mui et al. used radiolabeled ionizable lipids and PEG-lipids to independently track LNP and PEG-lipid fate in vivo and demonstrated that increasing PEG-lipid acyl length may decrease desorption rates [31]. As acyl length increased from C14 to C16 to C18, LNP half-life correspondingly increased from 0.64 h to 2.18 h to 4.03 h. In tissue-level biodistribution, C14 PEG-lipid LNPs rapidly accumulated in the liver, with 55% of the injected dose detected within 4 h, but exhibited divergent biodistribution between lipid components, consistent with partial dissociation of the PEG-lipid in circulation. In contrast, C16 and C18 PEG-lipid LNPs maintained closely matched lipid biodistribution profiles, consistent with reduced shedding [31]. Together, these findings demonstrate that acyl length-dependent PEG-lipid shedding is associated with differences in both pharmacokinetics and biodistribution of LNPs in vivo.

At the mechanistic level, these differences reflect a dynamic partitioning of PEG-lipids across multiple states rather than a simple on-off desorption process. Wilson et al. quantified shedding of C14 and C18 PEG-lipids from LNPs in serum using NMR spectroscopy [12]. After 12 h, 35% of C14 PEG-lipids had dissociated, whereas C18 PEG-lipids remained largely associated with the particles. Three populations were observed with distinct diffusion coefficients including LNP-associated PEG-lipids, free PEG-lipid unimers, and PEG-lipid micelles, which spontaneously form from free PEG-lipids [12]. More recent studies have reported similar findings with C18 PEG-lipids associating strongly with LNPs [32, 33]. These findings suggest that PEG-lipid shedding proceeds through a dynamic equilibrium between nanoparticle-bound and self-assembled states, with acyl length appearing to shift this equilibrium toward retention or release.

After loss of PEG-lipid, the colloidal stability of LNPs may begin to become compromised, leading to particle restructuring. Gallud et al. characterized these structural changes using small-angle neutron scattering and observed 3–5 nm reductions in polar radii and 0–3 nm increases in equatorial radii, indicating LNPs became more elliptically shaped for both C14 and C18 PEG-lipids [32]. Significant volume reductions were found to be around 30–40% for both formulations. The similar structural changes observed for both formulations might suggest comparable PEG-lipid loss. However, because the PEG layer of a typical LNP is approximately 3.5 nm thick [34], these changes could also arise from rearrangement or collapse of the PEG corona rather than complete PEG-lipid desorption. Consequently, structural characterization alone cannot distinguish PEG-lipid shedding from PEG rearrangement, highlighting the need for direct measurements of PEG-lipid dissociation.

These molecular differences may propagate to tissue-level outcomes, where acyl length-dependent shedding has been proposed as a strategy to tune organ distribution [30, 35, 36]. Waggoner et al. formulated LNPs with both C14 PEG-lipids and C18 PEG-lipids in a traumatic brain injury mouse model and found that increased accumulation in the brain and spleen correlated with increased C18 content [30]. Similar trends were reported when an ApoE peptide was bound to a C18 PEG-lipid to facilitate crossing the blood-brain barrier by exploiting the natural lipid uptake pathway of brain cells [37]. In these studies, LNPs contained 10 mol% C14 PEG-lipid or 10 mol% functionalized C18 PEG-lipid. The C18 LNPs exhibited significantly increased accumulation in both the brain and spleen, and it remains unclear whether these effects were driven by altered PEG-lipid shedding kinetics, ApoE-mediated targeting, or a combination of both. This highlights a broader challenge in the field where multiple interacting parameters are frequently changed simultaneously, limiting mechanistic insights and direct attribution of biological outcomes.

At the cellular level, reduced PEG-lipid shedding associated with longer acyl chains may delay or limit productive nanoparticle-cell interactions. Xu et al. found that C18 PEG-lipids prolonged uptake kinetics and reduced loading onto the RNA-induced silencing complex in hepatocytes compared to C14 PEG-lipids, illustrating how reduced shedding may alter downstream biological processing, although shedding was not directly measured in this study [38]. Similarly, Zhang et al. observed that C18 PEG-lipid LNPs exhibited reduced endosomal escape and translation efficiency across multiple in vitro and in vivo readouts [39]. In contrast, shorter acyl chains promoted more rapid functional delivery, including enhanced lymph node expression in vivo for C8 formulations [39]. Although differences in head-tail linkage in this study confound strict attribution to acyl length, the overall trend is broadly consistent with the hypothesis that prolonged PEG retention can sterically hinder membrane interactions, delaying uptake and limiting the intracellular processes required for effective endosomal escape [23, 40].

Taken together, acyl length appears to define a central design tradeoff in LNP engineering by coupling PEG-lipid anchoring strength to both systemic stability and intracellular delivery efficiency. Longer acyl chains are associated with more stable PEG-lipids that enhance circulation and reduce rapid, off-target uptake, but can impair cellular internalization and intracellular delivery (Figure 2). This tradeoff reflects the underlying kinetic competition between PEG-lipid desorption, nanoparticle clearance, and cell interaction. Designing optimal systems therefore requires balancing reduced clearance with sufficient uptake and endosomal escape to achieve functional delivery and matching PEG-lipid residence time to the desired biological context. However, a key unresolved challenge is the inability to decouple PEG-lipid shedding from other formulation-dependent and biological variables, which continues to limit precise mechanistic attribution and predictive design. To concretely establish the relationship between acyl chain length and PEG-lipid retention, future studies should systematically compare PEG-lipids spanning short, intermediate, and long acyl chains using direct measurements of shedding.

#### 2.1.2 | Branching

Lipid branching introduces an additional structural dimension that modulates PEG-lipid anchoring. Similar to acyl length, more branching results in higher van der Waals interactions between the lipid tail and the LNP, leading to stronger anchoring and reduced PEG-lipid shedding [12]. However, compared to acyl chain length, the effects of lipid branching on PEG-lipid shedding remain largely unexplored.

Experimental studies using dendritic PEG-lipids in siRNA LNPs suggest that increasing branching generation reduces delivery efficiency by limiting the shedding required for intracellular processing. Zhou et al. found that first-generation (G1) lipids achieved effective knockdown at substantially lower doses than their second-(G2) and third-generation (G3) counterparts in vitro [41]. Consistent with these findings, G2 and G3 lipids exhibited reduced delivery efficiency and failed to decrease murine tumor size in vivo. Mechanistic studies suggest that more highly branched PEG-lipids were unable to undergo endosomal escape efficiently. In contrast, less branched PEG-lipids dissociated from LNPs more readily, enabling nanoparticle destabilization, ionizable lipid protonation, and subsequent endosomal escape [41]. In this context, reduced shedding is associated with diminished functional delivery, reinforcing the idea that controlled PEG-lipid desorption is necessary for productive intracellular trafficking.

Notably, this trend is opposite to what has been reported for ionizable lipids, where increased branching enhances ionization and promotes endosomal escape [42–45]. This contrast highlights that the functional role of branching is context-dependent, with opposing effects depending on whether it is incorporated into the PEG-lipid or ionizable lipid component. As a result, branching cannot be considered an intrinsically beneficial or detrimental feature, but rather a parameter whose impact is defined by its role in regulating specific molecular transitions such as PEG-lipid desorption.

While increased PEG-lipid branching may reduce functional delivery by limiting shedding and intracellular processing, it may also confer advantages by stabilizing the nanoparticle and prolonging circulation. However, unlike acyl chain length, where the relationship between anchoring strength and in vivo behavior is relatively well defined, the impact of PEG-lipid branching on pharmacokinetics and biodistribution remains poorly characterized. Although branching likely modulates the same fundamental balance between nanoparticle persistence and delivery efficiency, its effects remain less predictable and less generalizable. Addressing this gap will require systematic studies that decouple branching from other design parameters and directly quantify its impact on PEG-lipid shedding in vivo, enabling its use as a more precise lever to tune LNP performance.

#### 2.1.3 | Saturation

The degree of saturation in PEG-lipid acyl chains is thought to modulate membrane packing, fluidity, and phase behavior, which may influence PEG-lipid mobility and shedding. Unsaturated lipids introduce kinks in the acyl chains that disrupt tight packing, increasing membrane disorder and weakening hydrophobic interactions, whereas saturated lipids promote more ordered and tightly packed bilayers that stabilize PEG-lipid anchoring. Consistent with this, an in vitro study comparing saturated PEG-DSPE and unsaturated PEG-DOPE found that unsaturated PEG-lipids resulted in increased antisense oligonucleotide delivery [7]. Similar to Zhou et al., the authors posited that the unsaturated bond allowed for more shedding from the particle, resulting in better endosomal escape, although shedding was not quantified in this study [7, 41].

Computational and experimental studies further support a packing-mediated mechanism of PEG-lipid shedding. Modeling efforts show that unsaturated PEG-lipids exhibit reduced packing and increased bilayer disorder, which can accelerate nanoparticle destabilization and payload release [46–48]. Experimentally, LNPs containing PEG-lipids with four unsaturated bonds released 33% of their payload over 7 days, compared to 16% for LNPs with two unsaturated bonds [49]. These findings may link decreased packing directly to increased release kinetics, supporting the role of saturation in governing nanoparticle stability.

At the membrane level, increased disorder also enhances PEG-lipid diffusion, which can facilitate desorption. Fluorescence recovery after photobleaching (FRAP) studies in small unilamellar vesicles (SUVs) revealed that PEG-lipids diffuse more rapidly in unsaturated DOPC membranes than in saturated DPPC membranes [50]. In FRAP, a region of the SUV is photobleached, and the recovery in fluorescence over time indicates the ability of PEG-lipids to diffuse within the membrane. Faster recovery corresponds to higher mobility, while slower recovery indicates restricted diffusion. In DOPC SUVs, unsaturated PEG-DOPE diffused more freely than saturated PEG-DSPE, whereas in DPPC SUVs both PEG-lipids were largely immobile [50]. These results demonstrate that reduced membrane packing enhances PEG-lipid mobility, which may facilitate desorption.

Consistent with this interpretation, membrane order also constrains PEG-lipid insertion and exchange processes. Post-insertion experiments, which are commonly used to incorporate targeting ligands after nanoparticle formation, revealed that saturated PEG-DSPE inserts less efficiently into saturated DSPC LNPs (~70%) than into unsaturated POPC LNPs (~80%) [51]. Similarly, PEG-DSPE inserted less efficiently than PEG-cholesterol derivatives into POPC LNP [51]. These findings further support the idea that tightly packed membranes restrict PEG-lipid mobility, potentially limiting both insertion and desorption.

Recipient cell membrane fluidity may also play a role in PEG-lipid mobility and particle-cell fusion. Membrane fluidity has been shown to vary substantially across cell types, and these differences likely affect LNP fusion and cargo delivery [52]. For example, T cells are known to have tightly packed, highly ordered lipid membranes that protect them from perforin-mediated cell lysis and may similarly restrict fusion with LNPs [53, 54]. Consistent with this idea, ex vivo treatment of PEG-lipid grafted polymeric particles showed that saturated PEG-DSPE intercalated into a lower proportion of T cell membranes (~0%) as opposed to B cells (~30%), dendritic cells (~70%), and macrophages (~50%) [55]. Conversely, PEG-DOPE intercalated into a greater proportion of T cells (~50%) and nearly all B cells, dendritic cells, and macrophages (~90–100%), likely because its unsaturated acyl chains disrupt lipid packing and facilitate cell membrane insertion. Likewise, increasing the fluidity of the nanoparticle membrane by increasing DOPE phospholipid content and decreasing cholesterol enhances mRNA delivery to primary human T cells [56]. These findings appear to broadly translate to in vivo systems, where lipid saturation influences liposome delivery [57]. He et al. found that incorporation of DOPE increased liposome fluidity and enhanced uptake by dendritic cells [57]. These studies suggest that the fluidity of both recipient cell membranes and delivery vehicles play a role in membrane fusion and downstream cargo delivery.

Together, these studies suggest that saturation influences PEG-lipid shedding through its control of membrane packing and fluidity (Figure 3). Highly ordered, saturated membranes appear to restrict PEG-lipid mobility and stabilize anchoring, whereas disordered, unsaturated membranes are associated with greater mobility and shedding. However, the functional consequences of these effects are inherently context-dependent. Increased membrane order has, in some cases, been associated with enhanced mRNA expression, potentially due to improved membrane fusion during cellular uptake, whereas increased fluidity may favor intracellular processing and cargo release [58]. Rather than defining a single optimal state, these findings indicate that saturation may modulate multiple, competing steps across the delivery cascade, including nanoparticle stability, cellular uptake, and intracellular trafficking.

Beyond bulk membrane properties, saturation may also influence membrane phase behavior, introducing an additional layer of complexity [59]. Unsaturated lipids such as DOPC preferentially associate with other unsaturated components, while saturated lipids such as DSPC form more ordered domains. This phase separation can affect the spatial distribution and density of surface-displayed ligands, which in turn influence cellular interactions and uptake [59–61]. Although these effects have not been directly examined for PEG-lipids, they suggest that saturation may regulate shedding not only through bulk fluidity but also through nanoscale organization within LNP membrane.

Similar to PEG-lipids, the saturation state of ionizable lipids modulates membrane fluidity and delivery efficiency. Inclusion of unsaturated bonds in ionizable lipids has been associated with improved endosomal escape and therapeutic delivery [62–66]. However, optimal performance requires balancing stability and fluidity, as excessive disorder could compromise nanoparticle integrity [64–67]. A similar design approach may be required for PEG-lipids, where the degree of saturation must be tuned to balance shedding, stability, and functional delivery. Uncovering the role of saturation in shedding will require investigation of PEG-lipids with varying degrees of unsaturation to build quantitative models of their effect on shedding.

Table 1 summarizes the effects of lipid chemistry on PEG-lipid shedding and nanoparticle targeting. Shedding dependency on acyl length has been directly quantified using methods including H-NMR, radiolabeled PEG-lipids, and size exclusion chromatography. However, the effects of other lipid chemistries, including branching and saturation, on PEG-lipid stability are commonly inferred from fluorescent readouts, biological outcomes, and computational modeling. In order to rigorously test the effects of variable lipid chemistry, more statistical, quantitative methods are needed.

## 3 | Head–Tail Linkage

The head-tail linkage between the hydrophilic PEG and hydrophobic lipid tail is an additional design parameter that influences PEG-lipid shedding from LNPs by modulating both interfacial charge and chemical stability. Common linkages include phosphates, carbamates, amides, and esters (Figure 4) [7, 35, 68–70]. Unlike lipid tail chemistry, which primarily tunes hydrophobic anchoring, linkage chemistry has been proposed to more directly influence PEG-lipid shedding by controlling surface charge and the mode of PEG detachment.

### 3.1 | Charge

Linkage-dependent charge can significantly alter PEG-lipid shedding and payload retention by modulating electrostatic interactions at the nanoparticle interface. For example, phosphate-containing PEG-DSPE carries a negative surface charge that has been associated with enhanced protein adsorption and accelerated drug release compared to neutral PEG-ceramide. The retention half-lives of the cationic drug vincristine are correspondingly shorter for PEG-DSPE than PEG-ceramide both in vitro (101 h vs. 156 h) and in vivo (23.9 h vs. 33.0 h) [68]. These differences are attributed to electrostatic attraction between the anionic lipid and cationic drug, which promotes drug migration toward the nanoparticle surface and facilitates release.

In parallel, increased protein adsorption to anionic PEG-lipids may accelerate PEG-lipid shedding [68]. Consistent with this hypothesis, Lu et al. found that the protein coronas of anionic PEG-DMPE and neutral PEG-DMG LNPs differed significantly [71]. The corona of PEG-DMPE contained more immunoglobulins, whereas PEG-DMG preferentially recruited apolipoproteins and coagulation factors [71]. These differences in protein corona composition were accompanied by enhanced in vitro and in vivo transfection of PEG-DMPE LNPs, which the authors attributed to increased nanoparticle destabilization and PEG-lipid shedding, although shedding was not quantified in this study [71]. Sarode et al. also showed that negatively charged PEG-DMPE achieved 2-fold greater gene silencing than neutral PEG-DMG, consistent with enhanced PEG-lipid desorption [7]. However, neutral PEG-ceramide outperformed negatively charged PEG-DSPE, achieving 1.5-fold greater gene silencing. These results suggest that charge alone cannot explain linkage-dependent efficacy and that the chemical structure of the linkage may influence PEG-lipid shedding and nanoparticle destabilization. In particular, carbamate-containing ceramide linkages may enable more favorable desorption kinetics, enhancing cellular uptake and endosomal escape [7, 72]. Independent studies of PEGylated lipoplexes have similarly reported improved siRNA delivery with PEG-ceramide compared to PEG-DSPE, which is consistent with the idea that linkage chemistry beyond surface charge may play a central role in governing PEG-lipid shedding and functional delivery [73].

### 3.2 | Degradability

In addition to charge, linkage degradability provides a second, orthogonal mechanism for modulating PEG-lipid shedding. Hydrolyzable linkages allow cleavage between PEG and the lipid tail, enabling rapid PEG detachment from the nanoparticle surface independent of desorption. Ester linkages are often rapidly hydrolyzed, while amides result in more variable detachment [35, 70, 74]. Carbamates exhibit superior stability during circulation while also maintaining efficacious delivery, which has been attributed to their relative resistance to hydrolysis and enzymatic degradation [75–77]. As a result, cleavage-prone PEG-lipids can accelerate PEG removal and promote nanoparticle destabilization, while more stable linkages prolong PEG retention. Binici et al. demonstrated that ALC-0159, a PEG-lipid with an amide linkage, produced greater mRNA expression than PEG-DMG, which contains an ester linkage, suggesting that linkage chemistry may influence endosomal escape and delivery efficiency, although this was not directly assessed [78]. This difference was only observed for LNPs formulated with ionizable lipid ALC-0315 as opposed to those formulated with DLin-MC3-DMA and SM-102. Optimization of these formulations, such as by modulating phospholipid, cholesterol, and PEG-lipid content and identity, could yield similar performance. This further emphasizes the need to consider how the composition of formulation-level factors can affect shedding.

The balance between cleavage and desorption is particularly important for optimizing delivery. Lee et al. showed that PEG-DOMG, a C14 PEG-lipid with a cleavage-resistant ether linkage, exhibited reduced siRNA delivery in vitro, an effect attributed to limited PEG-lipid shedding, along with prolonged circulation time and tumor accumulation in vivo compared to PEG-DMG [35]. Because ether and ester linkages have minimal effects on membrane permeability, these differences are attributed primarily to their distinct cleavage properties [79]. This highlights the idea that linkage chemistry can decouple circulation behavior from intracellular delivery, potentially by shifting the dominant mechanism of PEG removal.

Linkage chemistry may also have important safety implications by influencing the duration of PEG exposure and the fate of the detached PEG-lipid. More stable linkages prolong PEG retention on the nanoparticle surface, whereas hydrolyzable linkages promote more rapid PEG removal through bond cleavage. These differences could alter protein adsorption, immune recognition, and tissue exposure, although systematic comparisons of linkage-dependent safety remain limited. Future studies evaluating how linkage chemistry affects immunogenicity, tolerability, and off-target accumulation will be important for guiding the rational design of PEG-lipids for clinical applications.

Taken together, these studies suggest that head-tail linkage chemistry influences not only the extent but also the mechanism and kinetics of PEG-lipid shedding. Hydrolyzable linkages such as esters enable PEG detachment through bond cleavage, whereas more stable linkages such as amides and carbamates rely primarily on desorption from the nanoparticle surface. Ether linkages resist cleavage and therefore depend almost entirely on desorption. Thus, linkage selection influences whether PEG removal occurs through chemical degradation, desorption, or a combination of both, shaping the temporal profile of PEG retention on the nanoparticle surface. Addressing this gap will require systematic studies that decouple charge from biodegradability to clearly delineate how each factor affects shedding.

## 4 | PEG Chain

While the lipid tail primarily influences the strength of PEG-lipid anchoring within the nanoparticle and the head-tail linkage largely determines the mechanism of PEG detachment, the hydrophilic PEG segment plays a central role in modulating interactions with the surrounding biological environment. Specifically, the strong affinity of PEG for water forms a highly hydrated layer at the nanoparticle surface, which creates a steric barrier that reduces protein adsorption [9]. The enthalpic penalty associated with displacing water molecules from the PEG layer further disfavors protein binding [9]. As such, PEGylation attenuates complement activation, coagulation, and non-specific protein and antibody interactions that would otherwise promote immune recognition and rapid clearance by the mononuclear phagocytic system [80, 81]. Despite these protective effects, a measurable degree of protein adsorption still occurs in serum conditions and can influence PEG-lipid stability and shedding [80, 82]. In this section, we examine how variations in PEG physicochemical properties modulate protein interactions and ultimately impact PEG-lipid shedding.

### 4.1 | Length

PEG length is a widely used strategy for modulating nanoparticle surface properties; however, accurately characterizing PEG length remains challenging due to limitations in quantifying PEG polydispersity and molecular weight distributions. As a result, many studies report nominal or average PEG molecular weights rather than the precise PEG distribution present on the nanoparticle surface. Despite this limitation, studies have consistently shown that longer PEG chains generally reduce protein adsorption, improve pharmacokinetics, and enhance accumulation in target tissues. As PEG molecular weight increases, the polymer transitions from a condensed “mushroom” regime, in which individual chains are relatively collapsed and isolated, to an elongated “brush” regime, in which chains are more extended and densely packed [83–85]. This architectural shift has direct implications for protein adsorption. Longer PEG chains in the brush regime provide a more hydrated and flexible steric barrier that more effectively excludes plasma proteins than shorter PEG chains in the mushroom regime, which offer less uniform surface coverage [84, 86]. As a result, increasing PEG length is generally correlated with reduced protein corona formation by enhancing steric repulsion and increasing the energetic cost of displacing water associated with the PEG layer.

These reductions in protein adsorption have downstream effects on nanoparticle clearance in vivo. By limiting opsonization, longer PEG chains decrease recognition by the mononuclear phagocytic system and prolong circulation time [83, 85, 87–89]. However, some studies suggest that increasing PEG molecular weight beyond 5 kDa may provide minimal additional reduction in protein adsorption [90]. At the same time, excessively long PEG chains can introduce steric barriers that hinder interactions with cell membranes and receptor-ligand binding, thereby reducing cellular uptake and target engagement [61, 82, 91]. Furthermore, longer PEG chains have also been associated with the production of anti-PEG antibodies and accelerated blood clearance upon repeated administration [92].

In addition to reducing the absolute amount of protein adsorption, increasing the PEG length has also been found to modulate the composition of the protein corona. Longer PEG chains have been found to enrich certain proteins, including immunoglobulins and complement factors, while depleting others such as fibrinogen and apolipoproteins [93]. These compositional differences may alter nanoparticle biological identity and interaction pathways, further influencing biodistribution and cellular uptake.

PEG length-dependent effects on pharmacokinetics and functional delivery provide indirect evidence of PEG-lipid shedding. Shorter PEG chains, which permit greater protein adsorption, may increase PEG-lipid desorption and facilitate interactions that promote nanoparticle uptake. In contrast, longer PEG chains are associated with reduced protein corona formation, which may prolong circulation while limiting shedding and uptake. In terms of circulation time, rats treated with PEG-DSPE containing 5 kDa PEG (PEG5000-DSPE) had approximately 3-fold higher blood PEG-lipid concentration after 1 h compared to PEG750-DSPE, consistent with prolonged retention of longer PEG chains [70]. Increasing PEG length also altered biodistribution, with ex vivo fluorescence imaging revealing greater splenic accumulation for longer PEG chains [70, 83]. However, minimal differences were observed among shorter PEG lengths (350, 500, and 750 Da), suggesting the existence of a threshold PEG length required to effectively suppress protein adsorption and modulate in vivo behavior [70].

In terms of LNP uptake and functional delivery, Liu et al. found that PEG5000-lipids with C16–C18 lipid tails resulted in lower mRNA expression than those with PEG2000, suggesting reduced uptake due to increased steric hindrance and altered protein interactions [58]. In contrast, PEG5000-DMG and PEG2000-DMG showed similar mRNA expression, suggesting that rapid desorption driven by the labile C14 lipid tail may override PEG length-dependent differences, although desorption was not quantified in this study [58]. These results highlight that PEG length influences uptake and shedding, but its effects can be modulated by lipid anchor stability, overall PEG-lipid chemistry, and other formulation-level differences.

Overall, PEG chain length is a key parameter that strongly influences PEG conformation and protein corona formation, which may in turn influence PEG-lipid shedding. While longer PEG chains generally reduce protein adsorption and prolong circulation, these same properties can diminish protein-mediated interactions that contribute to PEG-lipid shedding and cellular uptake. However, the relationship between PEG length and shedding remains largely inferred rather than directly quantified. Future studies should therefore move beyond correlations with biodistribution and uptake to directly measure PEG-lipid desorption across defined PEG lengths under controlled biological conditions. Such studies will help establish design rules for tuning PEG length to balance circulation stability, protein interactions, and targeted delivery efficiency in lipid-based nanoparticle systems.

### 4.2 | Branching

The effects of PEG branching are generally considered analogous to those of increasing PEG length, as greater branching can increase effective PEG density at the nanoparticle surface and enhance steric repulsion of proteins. As a result, PEG branching provides a strategy to modulate surface coverage and improve circulation time, which may influence target cell uptake [94–98]. However, unlike PEG length, branching alters PEG architecture without increasing molecular weight, offering a distinct means to tune surface properties.

A recent study by Xiao et al. found that LNPs with branched PEG-lipids exhibited improved circulation and enhanced target transfection compared to formulations containing less branched or linear PEG-lipids [99]. The PEG-lipids used in this study had equivalent molecular weights, indicating that the observed differences were likely attributable to PEG architecture as opposed to PEG amount. Increased branching was associated with a denser brush-like configuration and reduced anti-PEG antibodies, which are hypothesized to facilitate PEG-lipid shedding [33]. However, the authors concluded that the lipid anchor properties, rather than the PEG structure, dominated PEG-lipid desorption behavior [99]. Consistent with this interpretation, other studies have reported minimal effects of PEG branching on circulation time and cellular uptake [94, 100, 101]. These findings suggest that while PEG branching can modulate protein interactions, its influence on PEG-lipid shedding may be secondary to lipid anchor stability.

Beyond protein interactions, PEG branching can also influence nanoparticle transport in biological environments. Cahn et al. demonstrated that PEG branching increases nanoparticle mobility and diffusion through extracellular matrices and mucus [94]. This effect was attributed to reduced polymer entanglement and improved conformational flexibility during transport through crowded biological barriers. These properties may be advantageous for delivery to mucosal tissues, including the gastrointestinal tract, nasal cavity, and bronchial epithelium [94, 102]. Additionally, because targeting ligands are often conjugated to the terminal ends of PEG chains, increasing PEG branching may provide a means to increase ligand density and potentially improve multivalent interactions with target cells.

Despite these potential advantages, the overall impact of PEG branching on in vivo performance remains context dependent. While branching can reduce protein adsorption in some settings, it has not consistently translated into improved circulation or uptake, and in some cases may negatively affect pharmacokinetics (Figure 5) [100]. Furthermore, PEG branching has not been systematically evaluated in the context of PEG-lipid shedding, and direct quantification of shedding dependent on PEG chemistry is lacking. Future studies should directly quantify how PEG architecture influences the extent and kinetics of PEG-lipid desorption, particularly in relation to protein corona composition. It is equally important to disentangle the effects of length and branching from each other since both can influence PEG architecture. Establishing these relationships will be critical to determine whether PEG branching can serve as a reliable design parameter for tuning shedding and optimizing targeted delivery while minimizing anti-PEG antibody responses.

Table 2 summarizes the effects of PEG chemistry on PEG-lipid shedding and nanoparticle targeting. Similar to lipid chemistry, shedding has not been statistically or rigorously quantified when testing PEG chemistries.

## 5 | Mechanisms of Shedding

PEG-lipid shedding has been suggested to arise from a combination of intrinsic physicochemical processes and biologically mediated interactions that together influence PEG-lipid residence at the nanoparticle surface. At baseline, PEG-lipids can undergo spontaneous desorption through equilibrium-driven partitioning into free unimers or micellar assemblies, although this process is relatively slow in the absence of external factors. Fluorescence microscopy studies have demonstrated that ~70% of LNP-associated PEG-lipid remains at the particle surface after 3 h in saline buffer under continuous flow, while NMR failed to detect free PEG-lipid micelles in some formulations [103, 104]. In biological environments, however, nanoparticle stability may be significantly affected through interactions with proteins, lipoproteins, and cellular membranes which could accelerate shedding. Protein adsorption may disrupt the hydrated PEG layer and increase PEG-lipid mobility, while lipid exchange with circulating lipoproteins and cell membranes provides a sink that could promote PEG-lipid removal. Additional processes such as cellular uptake, membrane remodeling, and enzymatic activity can further perturb membrane structure and enhance desorption. Among these mechanisms, protein corona formation and the accelerated blood clearance phenomenon represent two of the most well-characterized drivers of PEG-lipid shedding in vivo.

### 5.1 | Protein Corona

The protein corona forms as proteins adsorb onto the nanoparticle surface, altering its size, charge, and biological identity [18]. This process has also been proposed to promote PEG-lipid shedding by disrupting the hydrated PEG layer and increasing PEG-lipid mobility within the membrane, which may facilitate desorption. In the case of endogenously targeted PEGylated nanomedicines, PEG-lipid shedding can be functionally exploited to enable protein adsorption and downstream targeting. For example, SORT LNPs achieve tissue-specific tropism through distinct protein corona compositions [23]. The protein coronas of SORT LNPs exhibit enrichment of ApoE, *β*2-glycoprotein I, and vitronectin, which facilitate preferential targeting to the liver, spleen, and lung, respectively. Disruption of these compositions, such as by pre-incubating spleen-targeted SORT LNPs with ApoE, abrogates targeting specificity [15, 23]. These observations provide strong support for protein-mediated PEG-lipid displacement and subsequent adsorption of specific proteins as a dominant mechanistic model. However, other processes including spontaneous desorption, enzymatic degradation, and immune activation may also contribute to shedding. While protein-mediated shedding can enhance targeting, it can also be detrimental to nanoparticle performance [105, 106]. Protein adsorption can shield targeting ligands from binding their receptors, limiting therapeutic delivery [105]. This phenomenon has been described for peptide-, antibody-, and protein-targeted nanoparticles [106–110]. Although direct measurements of PEG-lipid desorption induced by protein corona formation remain limited, numerous studies report reduced targeting efficiency and altered nanoparticle delivery, supporting a model in which protein adsorption perturbs PEG layers and promotes PEG-lipid exchange or removal.

Thus, the protein corona may play a dual role in PEG-lipid shedding by both enabling and interfering with nanoparticle targeting. On one hand, several LNP formulations rely on the adsorbed proteins for endogenous targeting functionality. Conversely, the protein corona may shield active targeting ligands and facilitate shedding of targeted PEG-lipids. A key challenge moving forward is to quantitatively link protein corona formation to PEG-lipid desorption kinetics, as most current studies infer shedding indirectly from changes in targeting, biodistribution, or nanoparticle performance. While serum proteins, including apolipoproteins and other corona components, can contribute to PEG-lipid displacement in selected systems, the relative contributions of protein adsorption, lipoprotein exchange, spontaneous desorption, and chemical cleavage likely depend on nanoparticle composition and biological context. Systematic approaches that simultaneously measure protein adsorption and PEG-lipid loss will be essential to establish when and how the protein corona regulates PEG-lipid shedding and to enable more predictable nanoparticle design.

#### 5.1.1 | Accelerated Blood Clearance and Anti-PEG Antibodies

In addition to these protein corona-mediated processes, immune responses elicited by PEGylated nanomedicines have been proposed to further influence PEG-lipid shedding and nanoparticle clearance through the accelerated blood clearance phenomenon. Although PEGylation was initially used as a strategy to limit protein adsorption and immune responses [111], there is a growing body of evidence showing that repeated administrations can facilitate accelerated blood clearance. The initial administration can induce production of IgM, IgG, and IgE anti-PEG antibodies with IgM serving as the primary mediator of accelerated clearance [9, 112–114]. Subsequent injections can amplify anti-PEG antibody responses, leading to opsonization, complement activation, and rapid clearance from circulation [9, 82, 111, 115]. Complement activation can also lead to the formation of membrane attack complexes that integrate into the lipid envelope of LNPs much like they do in viruses, which may destabilize membrane integrity and facilitate PEG-lipid shedding [116]. The magnitude of these effects on pharmacokinetics depends on many factors including interval between injections, PEG-lipid chemistry (e.g., PEG length, PEG density, and linkage chemistry), and route of administration [70, 115, 117–119]. In addition to altered pharmacokinetics, anti-PEG immune responses have been associated with hypersensitivity and anaphylactoid reactions [120, 121]. Understanding the interplay between PEG-lipid shedding and anti-PEG antibody induction is therefore critical for the safe and effective design of PEGylated nanomedicines.

PEG-lipid surface density has been widely investigated as a factor influencing accelerated blood clearance. Ishida et al. reported that both high and low PEG-lipid densities can prolong circulation through different mechanisms [122]. PEG-lipid-poor LNPs may avoid B cell activation due to insufficient PEG epitopes to stimulate anti-PEG antibody production, whereas PEG-lipid-rich LNPs may evade immune recognition through steric shielding [115, 122]. However, other studies have reported contrasting results. For example, Li et al. observed similar anti-PEG antibody titers in rats after an initial dose of LNPs with varying PEG densities, but a second dose revealed faster clearance for LNPs with higher PEG content, likely due to increased antibody binding sites [123]. Despite these conflicting findings, several studies have proposed increasing PEG density as a strategy to mitigate accelerated blood clearance.

In addition to PEG density, PEG architecture has been found to play a role in modulating immune recognition. One approach involves branching PEG chains to enhance surface packing and promote brush-like conformations that reduce antibody accessibility [99]. Xiao et al. demonstrated that LNPs with bottlebrush PEG-lipids exhibited reduced binding to existing anti-PEG antibodies and correspondingly generated significantly lower antibody titers in mice compared to those with linear PEG-lipids [99]. PEG dendrimers have also been found to prolong circulation, with first-generation branching reducing anti-PEG IgM production and preserving initial pharmacokinetics upon successive administration [101, 118]. These effects are consistent with reduced immune recognition, although it remains unclear whether PEG architecture directly modulates PEG-lipid shedding or primarily acts by limiting the anti-PEG immune response.

PEG molecular weight is another factor that influences accelerated blood clearance. Jiao et al. systematically varied PEG length in a priming dose (400–5000 Da) followed by a second injection of PEG2000 phospholipids [117]. Priming with longer PEG chains (2000–5000 Da) caused rapid clearance of the second dose and increased accumulation in the spleen and liver compared to shorter PEG chains. This effect correlated with significantly higher anti-PEG IgM production in animals primed with PEG1000, PEG2000, or PEG5000, suggesting that longer PEG chains more effectively induce anti-PEG antibodies. In contrast, when the dosing sequence was reversed such that the priming dose was fixed (PEG2000) and PEG length was varied only in the second dose, similar clearance profiles were observed across all groups [117]. Similar trends have been reported in other studies of LNP formulations and PEGylated micelles, consistent with the idea that PEG length modulates immune priming [92]. Mechanistically, these immune responses likely reflect differences in PEG surface density, protein corona formation, and PEG-lipid shedding kinetics, all of which influence antigen presentation and B cell activation. Together, these findings suggest that PEG length should be selected in the context of dosing regimen, as it can shape both initial circulation time and the magnitude of anti-PEG immunity upon repeat administration, which is particularly relevant for applications requiring multiple doses such as cancer therapy and vaccination [124, 125].

The rate and degree of PEG-lipid shedding have also been proposed to affect the severity of the accelerated blood clearance phenomenon. Suzuki et al. showed that LNPs formulated with rapidly shedding PEG-DMG induced significantly lower anti-PEG IgM production than those containing more stable PEG-DSG (Figure 6) [33]. Upon repeat dosing, mice primed with PEG-DMG LNPs maintained therapeutic efficacy and hepatocyte targeting, whereas those primed with PEG-DSG LNPs exhibited reduced efficacy and increased uptake by Kupffer cells [33]. These results suggest that rapid PEG-lipid desorption may limit PEG exposure and reduce B cell activation, whereas prolonged PEG retention may promote antibody production and subsequent accelerated clearance. Similar relationships between length of lipid tail and clearance rate have been described in other studies, further supporting a potential role for PEG persistence at the particle surface as a central regulator of immune priming [126, 127].

In addition to modulating PEG length and branching, incorporating cleavable linkages between the PEG chain and the hydrophobic anchor has been investigated as a strategy to mitigate accelerated blood clearance [70, 128]. Xu et al. reported that repeated injections of PEG-cholesterol derivatives containing cleavable ester bonds maintained circulation times comparable to those observed after a single administration [128]. Cholesteryl hemisuccinate (CHEMS) linkages were more effective than cholesteryl methyl carbonate (CHMC) linkages, potentially due to the presence of an additional ester bond that enabled more efficient PEG cleavage and shedding. However, a more recent study found that while PEG-CHMC reduced anti-PEG IgM production, it did not maintain prolonged circulation, highlighting the challenge of balancing sufficient PEG retention for pharmacokinetic performance with rapid PEG removal to minimize immune recognition [70].

Overall, the accelerated blood clearance phenomenon high-lights a critical interplay between PEG-lipid shedding, immune recognition, and nanoparticle clearance. While stable PEG-lipids may prolong circulation, they can increase PEG exposure and promote anti-PEG antibody production upon repeated dosing. In contrast, more labile PEG-lipids may reduce immune activation by accelerating PEG shedding, but at the cost of reduced circulation time. Defining how PEG-lipid chemistry governs this balance remains an important challenge. Future studies should directly quantify PEG-lipid shedding in the context of immune activation and extend these analyses to targeted LNP systems, where the interplay between shedding, protein corona formation, and antibody binding is likely to be even more complex. Such efforts will be essential for establishing design principles that enable safe, effective, and repeatable dosing of PEGylated nanomedicines.

Beyond accelerated blood clearance, PEG-lipid retention may also influence acute immune responses to PEGylated nanomedicines. PEG-containing products, including laxatives and PEGylated LNP-based vaccines, have been associated with hypersensitivity and anaphylactic reactions in a small subset of individuals [81, 129, 130]. Acute reactogenicity to PEGylated formulations has been linked to multiple immune mechanisms, including pre-existing or induced anti-PEG antibodies. Anti-PEG IgM and IgG antibodies can activate complement pathways, contributing to complement activation-related pseudoallergy, which has been reported following administration of PEGylated liposomal doxorubicin [131]. In contrast, IgE-mediated recognition of PEG has been implicated in immediate hypersensitivity reactions through basophil and mast cell activation [131–133]. Because PEG-lipid shedding may regulate the duration of PEG exposure and nanoparticle surface presentation, desorption kinetics may influence immune recognition and tolerability. However, the relationship between PEG-lipid chemistry, shedding kinetics, and acute immune responses remains incompletely understood and requires further investigation.

Table 3 summarizes current studies that examine protein coronaformation and the accelerated blood clearance phenomenon and their relationship to PEG-lipid shedding. While there is some literature characterizing how biological mechanisms induce shedding, systematic evaluation of a wider range of formulations in complex environments is needed.

### 5.2 | Future Directions

#### 5.2.1 | Effects of Administration Route on Shedding

The impact of the administration route on PEG-lipid shedding remains poorly understood. Common routes of LNP administration include intravenous (IV), subcutaneous (SC), and intramuscular (IM) delivery, each of which exposes nanoparticles to distinct biological environments that may affect PEG-lipid stability. For example, nanoparticles administered via the SC route must traverse the interstitial fluid, lymphatic vessels, and cellular barriers before reaching systemic circulation, which may increase the likelihood of PEG-lipid shedding compared to direct IV administration.

Consistent with these differences, administration route strongly influences LNP biodistribution. Di et al. compared IV, SC, and IM injection of LNPs in mice and found that IV administration resulted in rapid systemic distribution with accumulation in the liver as early as 1 h after injection [134]. In contrast, LNPs delivered through SC and IM injections remained primarily localized near the injection site, with slower systemic dissemination [134]. These differences in biodistribution suggest that nanoparticles delivered through SC and IM routes may experience prolonged exposure to extracellular proteins and cellular barriers, which could influence PEG-lipid shedding kinetics.

Administration route may also affect immune recognition of PEGylated nanoparticles. Münter et al. reported that SC injection produced over 4-fold higher anti-PEG IgM titers than IV or IM administrations, possibly due to lymphatic drainage [135]. However, other studies suggest that anti-PEG antibody production may be less dependent on administration route and instead reflect different clearance mechanisms. Experiments involving splenectomized mice with inhibited complement activation demonstrated that anti-PEG IgM production following IV administration depends on complement-mediated activation in the spleen [136]. In contrast, anti-PEG antibody responses were still found in mice following SC injection, suggesting that the draining lymph nodes may contribute to B cell activation in this context [136]. These findings suggest that both the duration and location of PEG exposure, which are influenced by shedding kinetics and particle transport, contribute to B cell activation.

Together, these studies suggest that administration route governs PEG-lipid shedding indirectly by modulating the microenvironment and residence time of nanoparticles prior to systemic distribution, though this relationship has not been directly measured. SC and IM delivery expose LNPs to more complex and prolonged extracellular interactions, which may accelerate PEG-lipid shedding before reaching circulation, with downstream consequences for immune recognition and delivery efficiency. These interactions may also dictate initial protein corona formation due to variable protein compositions and concentrations found at the injection site [137]. Quantitatively linking route-dependent transport and microenvironmental exposure to PEG-lipid desorption kinetics and immune activation will therefore be important for designing optimal dosing strategies for PEGylated LNP therapeutics.

#### 5.2.2 | Biological Environment and Shedding

The biological environment surrounding nanoparticles may influence PEG-lipid retention. VanKeulen-Miller et al. demonstrated that LNP uptake and mRNA translation vary throughout the cell cycle, with cells in the G2/M phase exhibiting greater uptake and transgene expression than cells in the S or G0/G1 phases across multiple cell types [138]. Although PEG-lipid retention was not evaluated, differences in nanoparticle uptake and intracellular processing throughout the cell cycle could plausibly influence PEG-lipid desorption. Consequently, diseases characterized by altered cell-cycle dynamics, such as cancer, may exhibit distinct PEG-lipid shedding behavior that could contribute to differences in nanoparticle performance.

Patient-specific factors may also influence PEG-lipid retention by altering the protein corona. Disease states can substantially change the composition of circulating proteins and other biological fluids, including altered glycosylation of plasma proteins [139] and changes in respiratory mucus composition [140]. These differences are expected to alter protein corona composition and are already being leveraged for nanoparticle-based diagnostics [141]. Other patient-specific factors, including pregnancy, menstrual cycle, and obesity, have also been proposed to influence protein corona formation and nanoparticle transport [18, 142–144]. However, whether these biological environments affect PEG-lipid desorption remains largely unexplored.

Overall, the biological environment encountered by LNPs is likely to influence PEG-lipid desorption through changes in cellular physiology and protein corona composition. Defining these effects across diverse biological contexts will be important for developing predictive models of PEG-lipid shedding and designing formulations with consistent performance across patient populations.

#### 5.2.3 | Formulation-Level Determinants of PEG-Lipid Shedding

Beyond PEG-lipid chemistry, other formulation components, including ionizable lipids, helper lipids, and cholesterol, are also likely to influence PEG-lipid retention by altering membrane properties. Ionizable lipids such as ALC-0315 and SM-102 adopt cone-shaped geometries that promote membrane fusion and endosomal escape [78, 145, 146], partially attributed to reduced lipid chain packing and increased membrane fluidity [42, 43, 147]. On the other hand, ionizable lipids such as D-Lin-MC3-DMA are more cylindrical and form comparatively rigid LNPs [78, 146]. The differences in ionizable lipid chemistry have been shown to influence LNP pharmacokinetics [148] and organ tropism [149–153], but the relationship to PEG-lipid shedding remains unknown. Systematic studies examining the combined effects of ionizable lipid identity and PEG-lipid chemistry on desorption kinetics will be important for establishing general design principles for LNP formulations.

Helper lipids also contribute to membrane organization and may therefore influence PEG-lipid retention. Common helper lipids such as DSPC, DOPC, and DOPE differ in acyl chain saturation and molecular geometry, which alter membrane packing, fluidity, and fusion behavior [145]. DSPC is fully saturated and forms relatively rigid, tightly packed membranes, whereas the unsaturated phospholipids DOPC and DOPE increase membrane fluidity, with DOPE additionally promoting membrane fusion through its cone-shaped geometry [154]. These differences have been shown to influence LNP stability, intracellular delivery, and biodistribution. For example, Zhang et al. reported that LNPs formulated with DSPC preferentially accumulated in the spleen, whereas DOPE-containing formulations exhibited greater liver accumulation [155]. These findings suggest that helper lipids influence LNP behavior by modulating membrane organization, lipid–lipid interactions, and particle stability [156]. However, whether these changes also alter PEG-lipid desorption remains unknown. Future studies should systematically investigate how helper lipid identity and composition interact with PEG-lipid chemistry to modulate desorption kinetics and nanoparticle biodistribution.

Cholesterol is a major structural component of LNPs that works together with helper lipids to regulate membrane organization, packing, and stability [145, 157]. By filling spaces between phospholipids, cholesterol promotes tighter lipid packing, enhances membrane stability under physiologically relevant conditions, and influences the physical properties of the LNP [158]. Accordingly, modulation of cholesterol content has emerged as a strategy to tune LNP rigidity and biological performance. However, the effects of cholesterol on delivery are highly formulation- and context-dependent. Increasing cholesterol content can enhance mRNA expression in some settings, as demonstrated by Kauffman et al. and Ball et al., who found increased hepatic mRNA expression with higher cholesterol content [159, 160]. Conversely, Billingsley et al. found that decreasing cholesterol content enhances mRNA expression in T cells [56], while Su et al. demonstrated that cholesterol was not required for mRNA expression in the liver and lungs [161]. These findings highlight that cholesterol influences LNP behavior through complex interactions with other formulation components and biological environments. Because cholesterol modulates lipid packing and membrane dynamics, changes in cholesterol content may also influence the mobility and retention of PEG-lipids within the LNP membrane. However, the effect of cholesterol content on PEG-lipid shedding remains poorly understood. Future studies should evaluate how cholesterol-mediated changes in membrane packing and lipid organization influence PEG-lipid retention across diverse LNP formulations.

PEG-lipid shedding should be considered in the context of the overall LNP formulation rather than PEG-lipid chemistry alone. Ionizable lipids, helper lipids, and cholesterol each influence membrane organization, packing, and stability, which may collectively regulate PEG-lipid desorption. Given the diversity of LNP formulations, it is important to distinguish effects arising from PEG-lipid chemistry from those resulting from interactions among other formulation components. Furthermore, formulation composition strongly influences protein corona formation, which can alter nanoparticle biodistribution and cellular uptake [56]. Future studies should employ systematic, multifactorial experimental designs to determine how PEG-lipid chemistry and formulation composition together regulate PEG-lipid shedding and protein corona formation, ultimately informing the rational design of PEGylated LNPs.

#### 5.2.4 | Engineering Alternatives to PEG-Lipids

##### 5.2.4.1 | Amphiphilic Materials.

Alternative amphiphilic materials may provide opportunities to attenuate PEG shedding and the accelerated blood clearance phenomenon by altering how PEG is anchored and presented at the nanoparticle surface (Figure 7). In polymeric nanocarriers, PEG chains are typically linked to hydrophobic polymer blocks rather than lipid anchors, which can alter PEG presentation at the nanoparticle surface and influence immune recognition. Shiraishi et al. demonstrated that production and binding of anti-PEG IgM depend on the identity of the hydrophobic block within PEG-based block copolymers [119, 162]. For example, nanocarriers containing poly(lysine-1,4,7,10-tetraazacyclododecane-1,4,7,10-tetraacetic acid-gadolinium) (poly(lys-DOTA-Gd)) hydrophobic blocks did not induce detectable anti-PEG IgM responses or accelerated blood clearance, whereas those containing poly(*β*-benzyl l-aspartate) (PBLA) generated anti-PEG IgM but maintained prolonged circulation [162]. Shorter hydrophobic chains further reduced anti-PEG IgM binding, suggesting that polymer chemistry influences PEG immunogenicity. These findings suggest that tuning amphiphile structure may modulate both PEG shedding and immune recognition, although the mechanisms linking polymer anchoring, PEG presentation, and immune activation remain incompletely defined.

Despite these potential advantages, polymer-based PEG carriers do not universally reduce anti-PEG immune responses. PEG-poly(lactic-co-glycolic acid) (PEG-PLGA) nanoparticles exhibit desirable biocompatibility and biodegradability and have been used to target neutrophils and natural killer cells [163, 164] but also induce anti-PEG IgM production and subsequent accelerated blood clearance [136, 165]. In a study by Greiner et al., mice primed with PEG-PLGA nanoparticles exhibited significantly reduced nanoparticle circulation compared with non-primed animals, implicating complement activation and antibody-mediated clearance mechanisms [165]. These findings indicate that simply replacing lipid anchors with polymeric hydrophobic blocks is not sufficient to eliminate PEG immunogenicity and highlight the need to more precisely define how PEG density, conformation, and accessibility govern immune activation.

Other amphiphilic polymer systems may provide additional opportunities to control PEG presentation and stability. Poly(ethylene glycol)-block-poly(propylene sulfide) (PEG-b-PPS) nanocarriers, for example, self-assemble into vesicular polymersomes that exhibit high membrane stability and oxidation sensitivity, enabling controlled cargo release in early endosomes or inflammatory environments [166, 167]. PEG-b-PPS systems have been widely investigated for delivery to dendritic cells and macrophages and have demonstrated promising performance in vaccine applications [166]. In one such study, we demonstrated that polymersomes displaying the receptor binding domain (RBD) of the SARS-CoV-2 spike protein on their surface elicited stronger neutralizing antibody responses compared with polymersomes containing encapsulated antigen [168]. These findings highlight how surface presentation of ligands can enhance B cell activation through multivalent epitope display. Because PEG chains themselves can serve as antigenic epitopes, these observations suggest that nanoparticle surface architecture and ligand presentation may influence anti-PEG immune responses. However, whether PEG chains or surface ligands on polymeric nanocarriers undergo desorption or shedding analogous to PEG-lipids in LNP systems remains largely unexplored. Investigating ligand stability on polymer-based nanocarriers may therefore reveal how amphiphile chemistry and surface architecture influence immune recognition and PEG retention.

In addition to polymer chemistry, nanocarrier morphology may also influence ligand stability and shedding. Amphiphilic block copolymers can self-assemble into a variety of nanoarchitectures including polymersomes, filamentous micelles, and spherical micelles [169]. These structures differ substantially in membrane thickness, fluidity, and stability, all of which may affect the retention of PEG chains or targeting ligands. Polymersomes, for example, form highly stable vesicular structures with polymeric bilayers that promote hydrophobic interactions and reduce membrane permeability [170]. In contrast, micelles often exhibit lower stability, potentially facilitating ligand desorption, premature drug release, and off-target effects [171]. Differences in nanocarrier morphology have also been shown to alter biodistribution and cellular uptake profiles [169, 172, 173]. We found that PEG-lipid shedding from synthetic nanocarriers depended on both anchor strength and nanocarrier morphology [55]. PEG-lipid chemistry dominates shedding behavior for strongly anchored PEG-lipids (i.e., PEG-DSPE and PEG-DOPE), whereas nanocarrier morphology is the primary determinant of shedding for weakly anchored PEG-lipids (i.e., PEG-stearate and PEG-DMG). These differences translated to distinct in vivo outcomes, with PEG-lipids grafted onto polymersomes and micelles exhibiting biodistribution profiles that diverged from those of their parent nanocarriers, reflecting differences in PEG retention, particle size, and clearance pathways. Notably, even covalent attachment did not universally ensure ligand retention: while ligands conjugated directly to PEG-b-PPS remained stably associated with polymersomes, they partially dissociated from micellar formulations, indicating that nanocarrier instability can override linker chemistry. Together, these findings support the concept that ligand retention is not solely dictated by attachment strategy, but instead emerges from coupled effects of anchoring chemistry and nanoscale architecture, among other factors.

##### 5.2.4.2 | Adsorbent Materials.

An alternative strategy to mitigate ligand shedding is to avoid covalent anchoring altogether and instead exploit stable adsorption to present surface ligands. This approach parallels endogenous targeting, which leverages nanoparticle physicochemical properties to recruit host proteins that form a protein corona and direct biodistribution [18, 23]. In contrast, linker-independent ligand presentation involves the deliberate adsorption of targeting proteins or antibodies onto nanoparticle surfaces prior to administration. While both strategies rely on surface interactions rather than covalent attachment, endogenous targeting leverages host-derived proteins formed in situ, whereas adsorption-based approaches provide more direct control over the identity and density of targeting ligands.

One promising example is polypropylene sulfone (PPSU) nanoparticles, which can irreversibly adsorb proteins while preserving their functional targeting conformation. Using this noncovalent binding strategy, Du et al. generated dual-targeted nanoparticles by co-adsorbing antibodies against Siglec-6 and Fc*ε*RI*α* receptors to modulate mast cell activation [174]. Both in vitro and in vivo studies demonstrated strong inhibition of mast cell degranulation and anaphylaxis [174]. Similarly, citrate-functionalized gold nanoparticles have been found to retain adsorbed antibodies even in the presence of competing serum proteins [175]. These systems suggest that adsorption-based ligand presentation may provide a route to stabilize surface functionality without relying on PEG-lipid anchoring, although the long-term stability of adsorbed layers under physiological flow and exchange conditions remains to be fully defined [176].

##### 5.2.4.3 | PEG Alternatives.

An alternative strategy to overcome PEG-lipid shedding and accelerated blood clearance is to replace PEG with hydrophilic polymers that provide comparable steric stabilization while minimizing PEG-associated immune responses. One promising alternative is poly(sarcosine) (pSar), a biodegradable polypeptoid with physicochemical properties similar to PEG. Like PEG, pSar forms a highly hydrated steric barrier through interactions with water, enabling stealth properties and biocompatibility. However, unlike PEG, pSar can undergo cellular metabolism, which may reduce long-term accumulation [177]. Recent studies have demonstrated the feasibility of replacing PEG-lipids with pSar-lipids in LNPs. For example, Nogueira et al. found that pSar-lipid LNPs improved transfection and translation efficiency [178]. They also found that pSar-lipid LNPs prolonged circulation and even reduced off-target immune stimulation compared to PEG-lipid LNPs [178]. Similarly, Kang et al. reported improved delivery efficacy with pSar-lipid LNPs and demonstrated that these formulations did not bind anti-PEG antibodies in mice, highlighting their potential to avoid PEG-mediated immune recognition [179].

Beyond complete replacement of PEG, hybrid hydrophilic polymer architectures have also been explored to retain the favorable properties of PEG while reducing recognition by anti-PEG antibodies. Maiti et al. synthesized pSar-PEG block copolymers and demonstrated minimal anti-PEG antibody binding by ELISA and biolayer interferometry, suggesting that incorporation of pSar can reduce immune recognition of PEG-containing materials [180]. In addition to LNPs, pSar-containing polymers have shown promise in other nanocarrier platforms. For example, pSar-PEG-PLGA nanoparticles exhibited improved pharmacokinetics and enhanced bioavailability of chemotherapeutic agents compared with conventional formulations, further supporting the potential of pSar-based hydrophilic coatings to improve nanoparticle performance [181].

Another class of alternative polymers being actively explored is poly(cyclic imino ether)s (PCIEs), including poly(oxazoline)s (pOx) and poly(oxazine)s (pOz). Similar to PEG and pSar, PCIEs interact with water through hydrogen bonding, enabling the formation of hydrated polymer coatings that can provide steric stabilization [182]. Unlike pSar, PCIEs are not biodegradable but offer a highly tunable chemical platform, with variations in polymer backbone and side-chain chemistry enabling control over nanocarrier properties [182, 183]. He et al. synthesized a series of pOx- and pOz-lipids, including poly(ethyl oxazoline) (pEtOx), poly(methyl oxazoline) (pMeOx), and poly(methyl oxazine) (pMeOz), and evaluated their effects on LNP delivery [184]. Compared with PEG-DMG LNPs, pEtOx-, pMeOx-, and pMeOz-containing LNPs improved Jurkat T cell transfection, highlighting the potential of PCIE-lipids as alternatives to PEG-lipids [184]. The highly tunable characteristics of these polymers may also influence pharmacokinetics and biodistribution. While He et al. observed similar biodistribution profiles among pEtOx, pMeOx, and pMeOz LNPs, other studies have demonstrated that subtle changes in polymer chemistry can produce distinct biological outcomes. Humphries et al. formulated polymeric nanoparticles using pEtOx and pMeOx and found that pEtOx nanoparticles exhibited reduced blood retention, increased lung accumulation at later time points, and greater uptake by antigen presenting cells compared with pMeOx nanoparticles [185]. Beyond altering biodistribution, Moreadith et al. demonstrated that LNPs formulated with poly(ethyl oxazine) (pEtOz)-lipids avoided binding by both anti-PEG IgM and IgG following repeated administration in vivo, highlighting their potential to reduce PEG-associated immune recognition [183].

Zwitterionic polymers represent another promising class of PEG alternatives. These materials contain both positive and negative charges within the same monomer, resulting in a net neutral charge. In contrast to PEG and pSar, which rely primarily on hydrogen bonding, zwitterions interact with water through strong ion-dipole interactions that generate a tightly bound hydration layer, reducing nonspecific protein adsorption and enhancing stealth properties [186]. Several groups have incorporated zwitterionic polymers into LNP formulations to improve delivery while avoiding PEG-associated immune recognition. For example, Khunsuk et al. developed LNPs with poly(2-methyacryloyloxyethyl phosphorylcholine)-lipids that demonstrated cytocompatibility and effective nucleic acid delivery in human cell lines [187]. Similarly, Luozhong et al. reported that poly(carboxybetaine)-lipid (PCB-lipid) LNPs improved liver transfection and gene-editing efficiency while avoiding anti-PEG antibody binding [188]. Beyond liver delivery, PCB-lipid LNPs have also been utilized to facilitate delivery to the lungs. Jiang et al. incorporated PCB-lipids to enhance the hydration shell surrounding LNPs, improving transport through airway mucus and increasing pulmonary mRNA expression [189]. Repeated administration maintained improved mRNA expression, suggesting that these formulations may avoid the accelerated blood clearance response associated with PEGylated nanocarriers [189]. Li et al. further demonstrated that PCB-lipid LNPs improved transfection of inflammatory macrophages, with protein corona analysis revealing enrichment of complement factor D and fibrinogen alpha chain, which may contribute to enhanced phagocytic uptake [190]. In addition to replacing PEG-lipids with alternative hydrophilic polymers, recent approaches have explored eliminating PEG entirely through the use of zwitterionic lipids. Zhao et al. developed a three-component LNP formulation containing a zwitterionic ionizable lipid capable of forming stable, spherical nanoparticles for mRNA delivery [191]. These LNPs exhibited splenic tropism in mice following repeated administration and improved survival in a melanoma model compared with the Pfizer-BioNTech LNP formulation [191]. These studies demonstrate the potential of zwitterionic materials to provide PEG-independent strategies for enhancing nanoparticle stability, reducing immune recognition, and enabling repeated administration.

Together, these studies highlight the potential of PEG-free nanocarrier designs that provide stealth-like properties and maintain delivery performance while avoiding PEG-associated immune recognition (Figure 8). By eliminating PEG-lipid conjugates altogether, these systems may circumvent PEG-specific desorption and reduce susceptibility to antibody-mediated clearance. However, key questions remain regarding how these alternative hydrophilic materials influence nanoparticle biodistribution, intracellular trafficking, and long-term immune recognition. In particular, future work should explore strategies for conjugating ligands to pSar-, PCIE- or zwitterionic-lipid surfaces and evaluate how these ligand-display methods affect surface stability, shedding behavior, and clearance. Systematic comparisons between PEGylated and PEG-free systems will be essential to determine whether these materials fully eliminate shedding-like phenomena or introduce new modes of surface instability, ultimately informing the rational design of next-generation nanocarriers with improved pharmacokinetics and targeting capacity.

## 6 | Techniques to Investigate Shedding

The majority of studies on PEG-lipid shedding rely on in vitro cell-based assays and in vivo mouse models, often without directly quantifying desorption in isolation from dynamic biological processes that can confound interpretation. This limitation primarily stems from the lack of robust, reproducible methods available for single-particle quantification [192]. Optical and non-optical techniques, which are widely used to study extracellular vesicles (EVs), offer a promising toolkit for nanoparticle characterization and are particularly well suited to probing dynamic changes in surface chemistry [193, 194]. These methods, including Förster resonance energy transfer (FRET), nano flow cytometry (NanoFC), nanoparticle tracking analysis (NTA), total internal reflection fluorescence (TIRF) microscopy, and Raman spectroscopy, provide complementary insights into PEG-lipid stability across length and time scales (Figure 9) [60, 192, 193, 195, 196].

FRET is one of the most accessible approaches for probing PEG-lipid shedding. In this method, nanoparticle components are labeled with donor and acceptor fluorophores that exhibit spectral overlap. Upon excitation of the donor, energy transfer to the acceptor produces a secondary emission signal that is highly sensitive to intermolecular distance (1–10 nm), enabling detection of nanoscale structural changes [197]. As such, FRET has been widely used to assess nanoparticle stability over time [198, 199]. Because energy transfer can be maintained across hydrophobic and hydrophilic domains, FRET is also applicable to structurally complex systems such as polymersomes. Together, these features make FRET well suited to infer PEG-lipid dissociation, where decreases in energy transfer are interpreted as increased spatial separation following desorption [200]. In our work, dual-labeled nanocarriers incubated in serum revealed that shedding depends on both PEG-lipid chemistry and nanocarrier morphology [55]. However, when measured using a conventional spectrophotometer, FRET inherently reports ensemble-averaged behavior and is therefore insensitive to particle-to-particle heterogeneity in shedding kinetics. In addition, changes in local membrane organization, probe distribution, or fluorophore orientation can influence FRET efficiency independent of PEG-lipid loss, complicating quantitative interpretation. Therefore, ensemble FRET should be paired with orthogonal methods, such as single-molecule FRET (smFRET), which measures energy transfer at the level of individual particles to resolve heterogeneity and validate mechanistic conclusions.

NTA enables single-particle characterization by tracking Brownian motion to estimate particle size, while simultaneously reporting fluorescence from labeled species [193]. In contrast to dynamic light scattering, which provides ensemble-averaged measurements, NTA resolves individual particles within a defined focal volume and can capture population heterogeneity. For PEG-lipid shedding, NTA is best suited to monitor decreases in particle-associated fluorescence following exposure to shedding conditions, which can provide evidence of loss of labeled PEG-lipid. As shedding progresses, however, the system also becomes increasingly heterogeneous and difficult to deconvolute. Furthermore, the apparent “visualization” provided by NTA reflects reconstructed particle trajectories derived from scattering signals rather than direct imaging, limiting mechanistic insight. As a result, NTA is most effective when used in combination with orthogonal methods to support conclusions regarding PEG-lipid desorption.

NanoFC extends conventional flow cytometry to the nanoscale, enabling high-throughput, single-particle measurements of size and fluorescence [201]. This approach has been used to quantify surface density of targeting ligands on nanoparticles and could be adapted to quantify PEG-lipid shedding [60]. However, accurate NanoFC measurements require tight control of particle concentration (10^7^–10^9^ particles/mL) [193], and the presence of serum lipoproteins within a similar size regime may necessitate rigorous purification prior to measurement [202]. Additionally, NanoFC provides endpoint measurements rather than real-time dynamics, limiting its ability to capture the kinetics of PEG-lipid shedding.

TIRF microscopy offers a powerful and complementary approach by enabling real-time visualization of PEG-lipid shedding at the single-particle level. In TIRF, excitation light directed at the glass-water interface above the critical angle generates an evanescent field that selectively excites fluorophores within ~100 nm of the surface [203]. This confined illumination minimizes background fluorescence from bulk solution, enabling high signal-to-noise imaging of surface-associated nanoparticles. Accordingly, TIRF has been widely used to study processes such as LNP fusion and endosomal escape on model lipid bilayers [204–207]. For studies involving serum-induced PEG-lipid shedding, the immobilization strategy is critical. Conventional neutravidin-biotin interactions used to bind LNPs to the surface of the cover glass [204, 208, 209] rely on surface-adsorbed neutravidin, which can undergo exchange or displacement by serum components, leading to LNP detachment and confounding results. More stable attachment strategies, such as covalent tethering by functional silane-PEG, can improve robustness while limiting nonspecific adsorption. Although TIRF provides high spatial and temporal resolution, it is lower throughput than flow-based methods and subject to photobleaching. In our work, we leveraged TIRF with click chemistry-based immobilization to quantify PEG-lipid shedding in real time, enabling extraction of dissociation constants that depend on both PEG-lipid chemistry and nanocarrier morphology [55].

Despite their utility, many optical techniques rely on fluorescent labels that may be altered under biologically relevant conditions, such as changes in pH or enzymatic degradation. Additionally, fluorescent tags may perturb interactions among LNP components or alter nanoparticle interactions with biological systems. Therefore, measurements obtained from fluorescently labeled systems may not fully capture native LNP behavior. Complementary approaches that minimize perturbative labeling are needed to characterize PEG-lipid shedding under more physiologically relevant conditions. Nuclear magnetic resonance (NMR) spectroscopy, for example, can distinguish intact nanoparticles, PEG-lipid micelles, and free PEG-lipids based on differences in diffusion coefficients [12]. Electron microscopy (EM) provides structural characterization of heterogeneous nanoparticle populations, while atomic force microscopy (AFM) enables nanoscale analysis of particle morphology and surface organization [210–213].

Scattering techniques, such as small-angle neutron scattering (SANS) and small-angle X-ray scattering (SAXS), have also been used to probe LNP membrane organization [214], structural changes associated with PEG-lipid shedding [32], and structural stability under varying salt and enzyme concentrations [215]. SANS offers the advantage of contrast variation, enabling selective visualization of individual nanoparticle components, although this often requires isotopic substitution and specialized sample preparation. In contrast, SAXS is more broadly accessible and does not require isotopic substitution but provides less component-specific information [214]. Together, these techniques are well suited to characterize how PEG-lipid shedding alters LNP structure. Future studies should move beyond simplified formulations and evaluate increasingly complex biological environments, including serum proteins, to better understand how PEG-lipid shedding influences LNP structure under physiologically relevant conditions.

While scattering techniques reveal nanoscale structural organization, they provide limited information regarding changes in molecular composition. Raman spectroscopy addresses this gap by probing the molecular vibrational signatures of lipids. Barriga et al. used Single Particle Automated Raman Trapping Analysis (SPARTA) to characterize changes in LNP composition and stability following phospholipase D treatment, demonstrating conversion of DOPC to DOPA through changes in characteristic Raman spectral features [215]. Louro et al. similarly applied SPARTA to characterize LNP-EV hybrids, revealing Raman spectral features consistent with differences in lipid composition and degree of unsaturation relative to EV controls [216]. Future studies should integrate Raman spectroscopy with complementary structural techniques to determine how PEG-lipid identity and formulation composition influence the molecular and structural changes that accompany PEG-lipid shedding.

No single method can fully capture the complexity of PEG-lipid shedding, making it essential to integrate complementary approaches across multiple spatial and temporal scales. Ensemble measurements reveal average behavior but mask particle-to-particle variability, while single-particle methods provide insights into heterogeneity and dynamics yet face limitations in resolution, throughput, or physiological relevance. Moreover, in vitro and in vivo studies alone cannot readily distinguish shedding from other biological processes, underscoring the need for label-free techniques that directly interrogate nanoparticle structure and chemistry. Moving forward, combining advances in super-resolution microscopy with complementary structural and compositional methods will be critical to quantify PEG-lipid shedding in situ and in real time while linking dynamic PEG-lipid desorption to changes in nanoparticle organization and composition. Such a multi-method, mechanistically informed framework will not only clarify the molecular drivers of PEG-lipid shedding but also guide the rational design of more stable and precisely targeted nanomedicines.

## 7 | Conclusions

Lipid-based nanomaterials have emerged as a versatile platform for the delivery of nucleic acids, proteins, and small molecules. Central to their performance are PEG-lipids, which are widely used to modulate interfacial properties and reduce immune recognition. However, PEG-lipids are not static; their dynamic dissociation in biological environments plays a defining role in shaping nanoparticle behavior in vivo. Rather than a secondary phenomenon, PEG-lipid shedding represents a key determinant of the balance between circulation, immune recognition, and cellular uptake. The factors that mediate this process can be broadly categorized into chemical determinants arising from PEG-lipid structure and membrane properties, and biological determinants mediated by interactions with the surrounding environment.

From a chemical perspective, PEG-lipid shedding is influenced by molecular features that control lipid insertion, retention, and exchange within nanoparticle membranes. Among these, lipid anchor length remains the most extensively studied parameter, with longer acyl chains generally reducing desorption. However, other important chemical determinants of shedding are comparatively underexplored. Structural features such as lipid branching, degree of saturation, and linkage chemistry are also expected to influence shedding by modulating membrane packing, phase behavior, and lipid mobility. In parallel, while the PEG segment has been widely optimized to reduce protein adsorption, its contribution to desorption kinetics remains less well defined. Additionally, other LNP components, including the ionizable lipid, helper lipids, and cholesterol, influence membrane organization and may therefore affect PEG-lipid retention. Taken together, these observations suggest that PEG-lipid shedding cannot be attributed to a single molecular parameter but instead emerges from the combined effects of lipid structure and membrane organization, which in turn influence interactions with biological environments. Accordingly, systematic evaluation of PEG-lipid chemistry within diverse LNP formulations will be essential to rationally optimize delivery to target cells and tissues.

Biological determinants further regulate PEG-lipid shedding through dynamic interactions at the nanoparticle interface. The formation of a protein corona introduces competing adsorption processes that can both stabilize and displace PEG-lipids, depending on protein identity and binding affinity. Consequently, the protein corona can facilitate nanoparticle trafficking, obscure targeting ligands, or promote clearance, thereby influencing nanoparticle fate in vivo. In addition, anti-PEG antibodies and the accelerated blood clearance phenomenon link PEG-lipid behavior to immune recognition, which is a key determinant of nanoparticle performance upon repeat administration. Emerging evidence suggests that PEG-lipids with faster desorption kinetics may reduce anti-PEG antibody generation, highlighting the complex interplay between PEG stability and immunogenicity. PEG-lipid shedding may also vary with patient-specific biological factors, including disease state and cellular physiology, further contributing to interindividual variability in nanoparticle behavior. Together, these observations demonstrate that PEG-lipid shedding is mediated not only by nanoparticle design but also by continuous exchange between the nanoparticle surface and its surrounding biological environment. Accordingly, future LNP formulations should be evaluated across diverse biological contexts to better account for patient-to-patient variability and support the development of more personalized nanomedicines.

A central challenge moving forward is the lack of quantitative frameworks to describe and predict PEG-lipid shedding. Current studies often rely on indirect or system-specific measurements, limiting comparisons across formulations and hindering the identification of generalizable design principles. The development of standardized, quantitative methods to measure PEG-lipid desorption kinetics, combined with systematic variation of lipid chemistry and nanoparticle architecture, will be essential for establishing robust structure-function relationships. Ultimately, PEG-lipid shedding introduces a fundamental design tradeoff. Rapid desorption can shorten circulation times and promote premature clearance, whereas persistent PEG layers may inhibit cellular interactions required for uptake and promote the formation of anti-PEG antibodies. Optimizing this balance will require moving beyond static design parameters toward strategies that account for the timing and extent of interfacial exchange. Approaches that tailor PEG-lipid chemistry, leverage route-specific biological environments, or incorporate alternative stealth materials may provide new opportunities to engineer nanoparticle behavior. As quantitative understanding of PEG-lipid shedding continues to improve, these strategies will enable the rational design of nanomedicines with enhanced stability, targeting, and therapeutic efficacy.

Figure 10 summarizes key opportunities to incorporate PEG-lipid shedding into the rational design and preclinical evaluation of PEGylated LNPs. These considerations highlight that PEG-lipid shedding should be viewed not as an unavoidable limitation, but as a controllable design parameter that can be leveraged to optimize nanoparticle performance. Continued integration of quantitative measurements with advances in nanoparticle engineering and a deeper understanding of biological interactions will enable the development of nanomaterials with tunable interfacial dynamics, improved therapeutic performance, and greater translational potential.

## Figures and Tables

**FIGURE 1 |:**
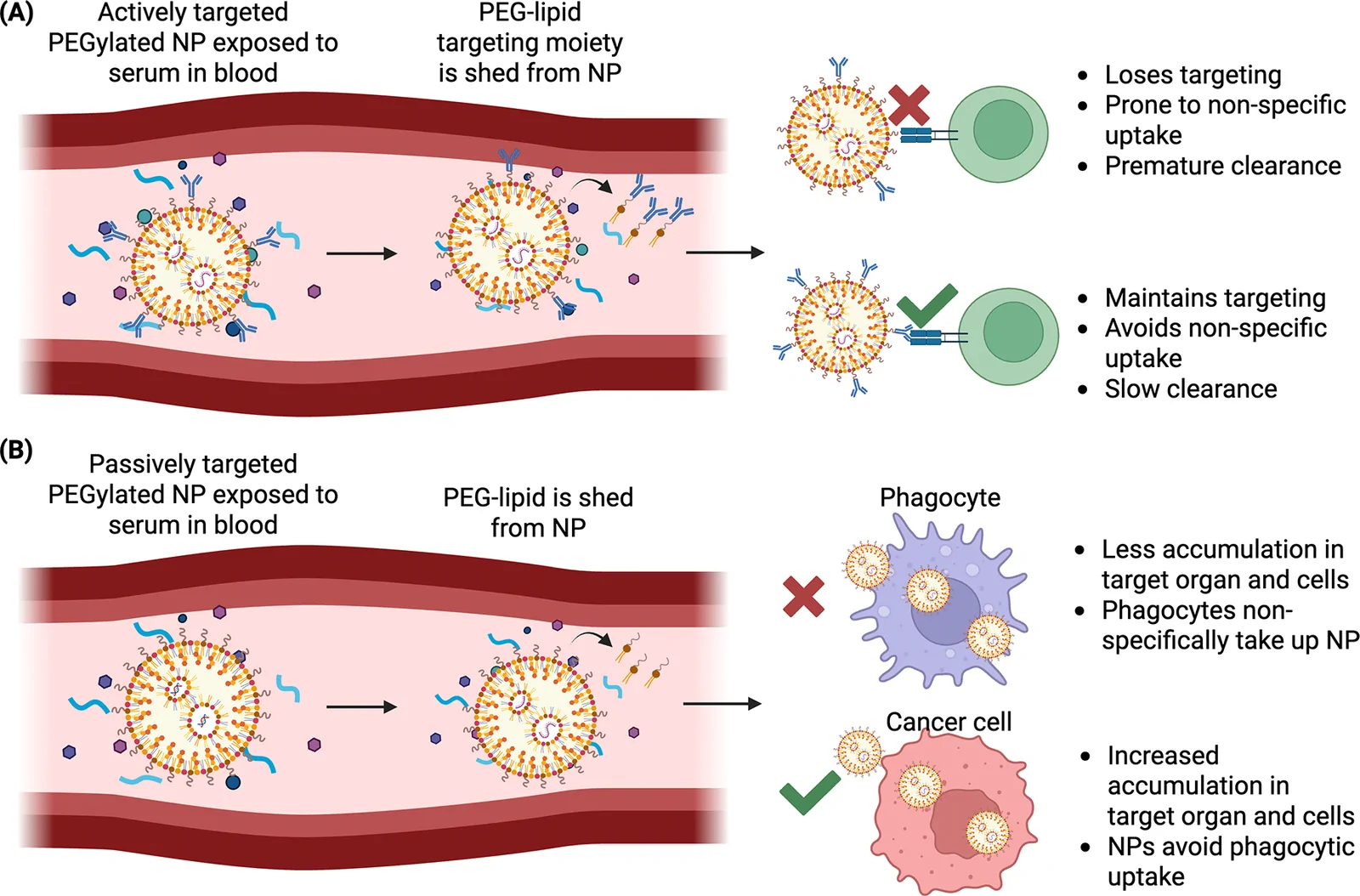
Loss of PEG-lipids can lead to less therapeutic delivery to target cells and organs in both (A) active targeting and (B) passive targeting strategies.

**FIGURE 2 |:**
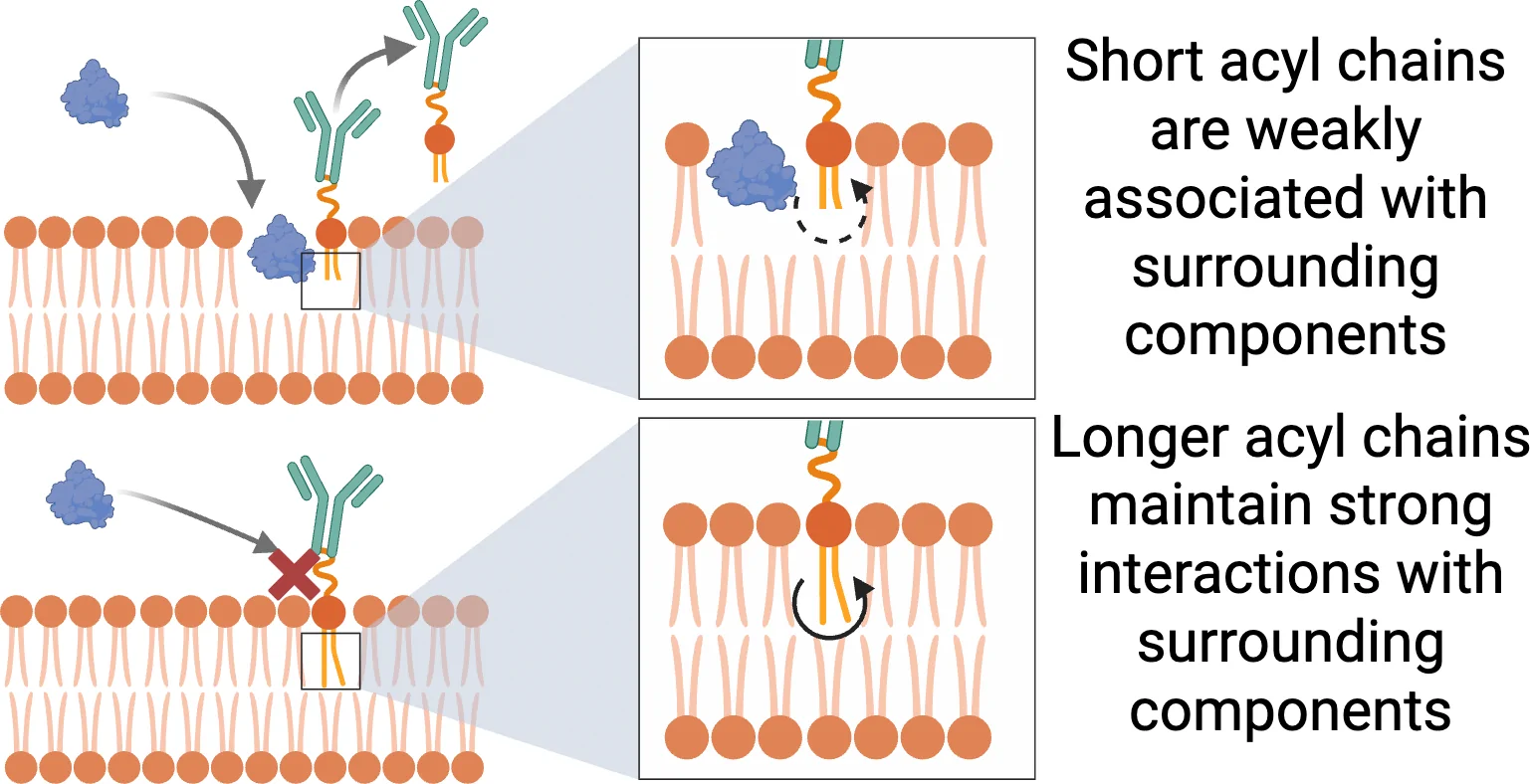
Acyl length affects shedding profile due to the strength of van der Waals interactions. Serum proteins may more readily extract PEG-lipids with shorter acyl chains, whereas longer acyl chains exhibit stronger membrane interactions that may resist protein-mediated extraction.

**FIGURE 3 |:**
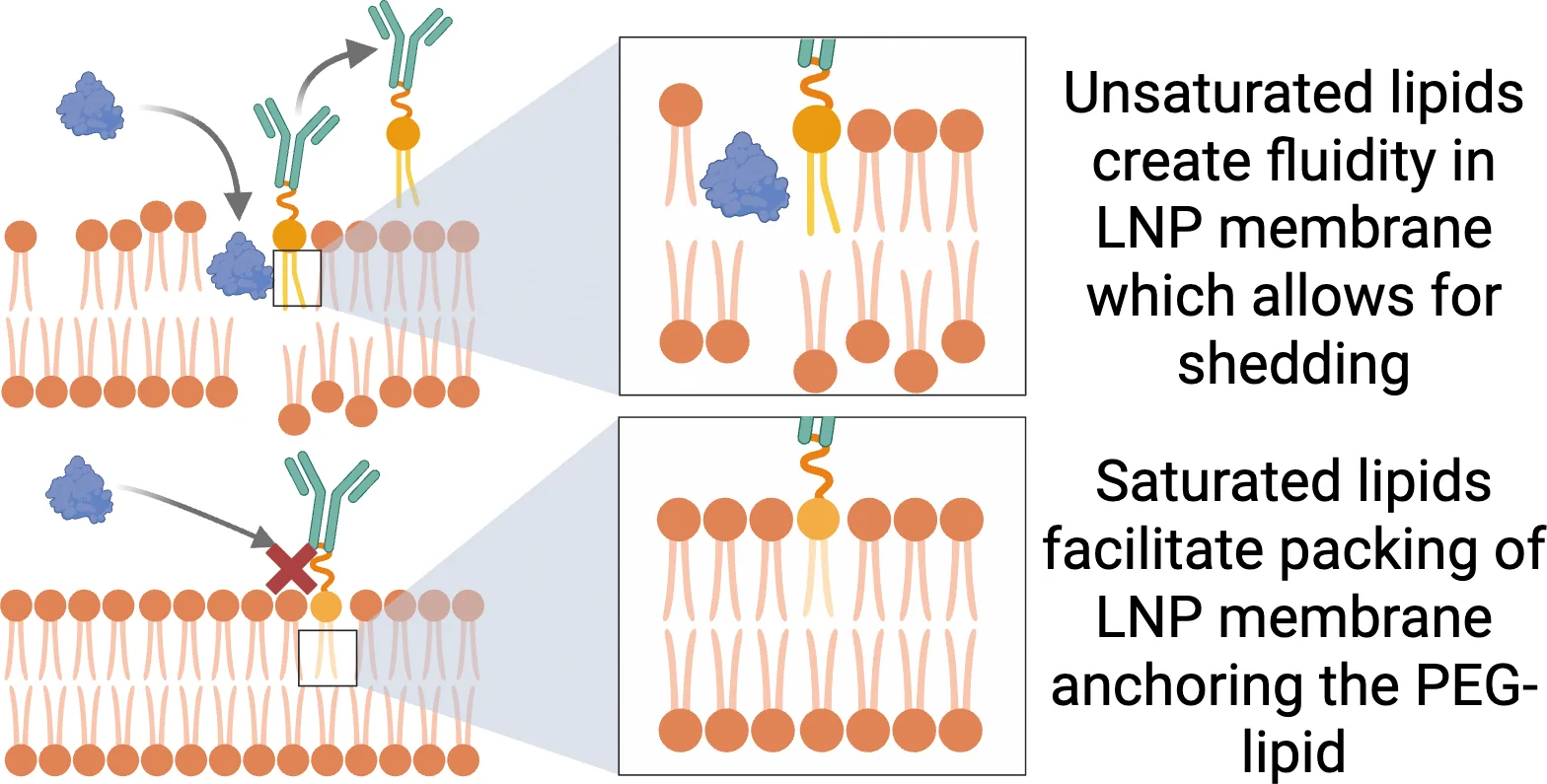
Saturation affects fluidity and packing of lipids in LNP membranes. In a membrane composed of saturated lipids, unsaturated PEG-lipids increase fluidity.

**FIGURE 4 |:**
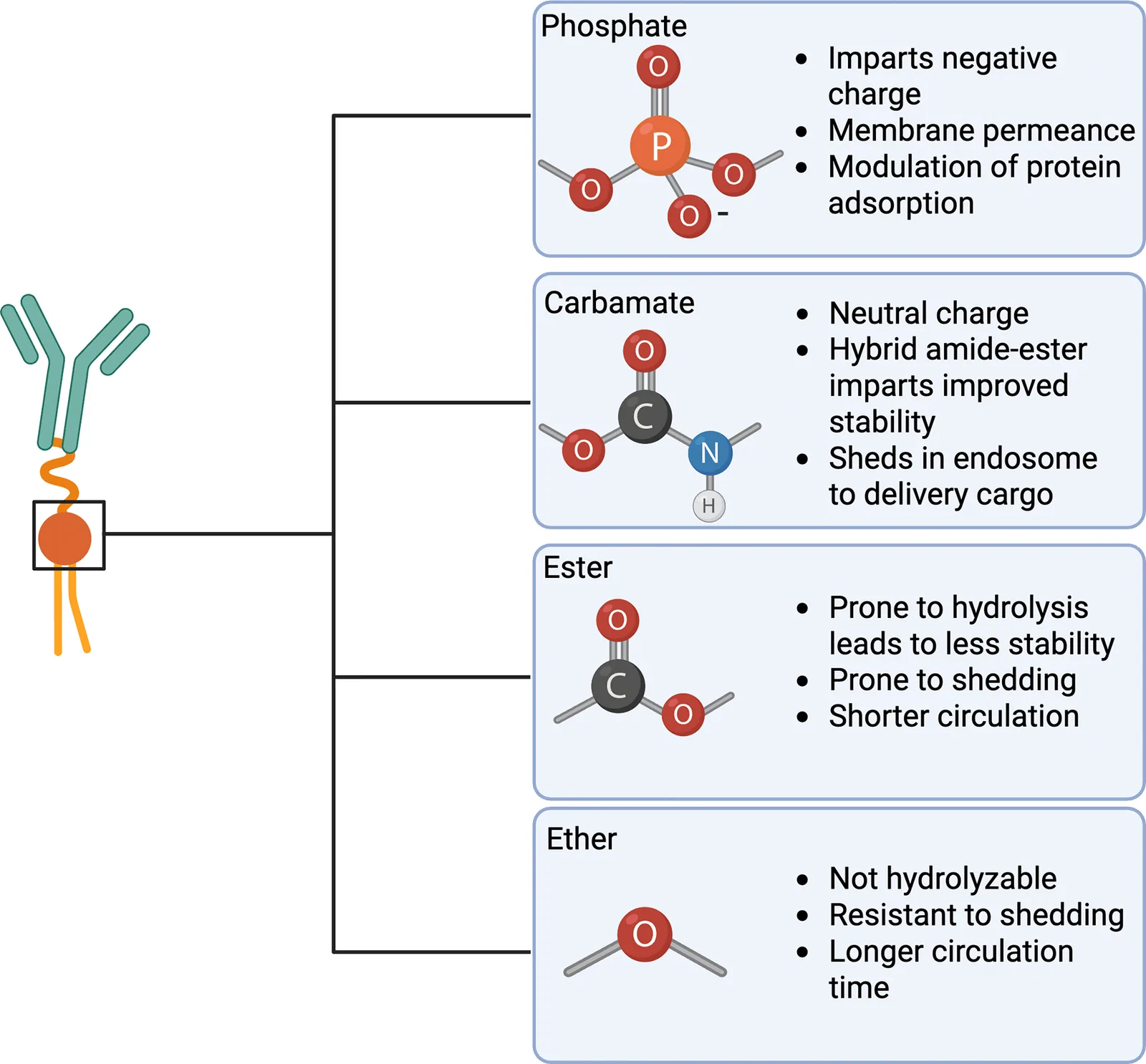
Head-tail linkages contribute to the shedding of PEG-lipids by affecting their stability and cleavability

**FIGURE 5 |:**
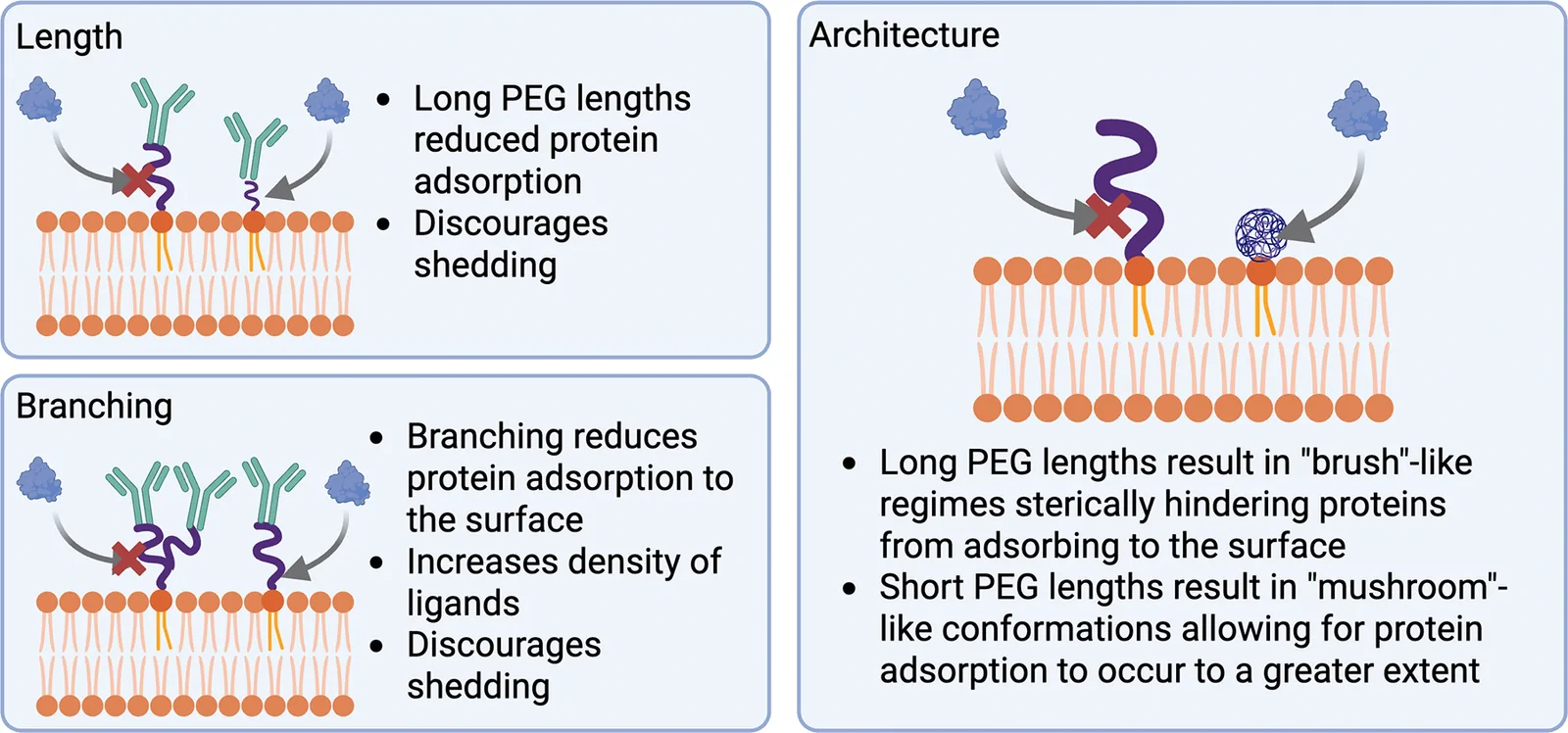
Increasing both length Repand branching lead to less protein adsorption. Branching could be a strategy to increase both PEG and targeting moiety density.

**FIGURE 6 |:**
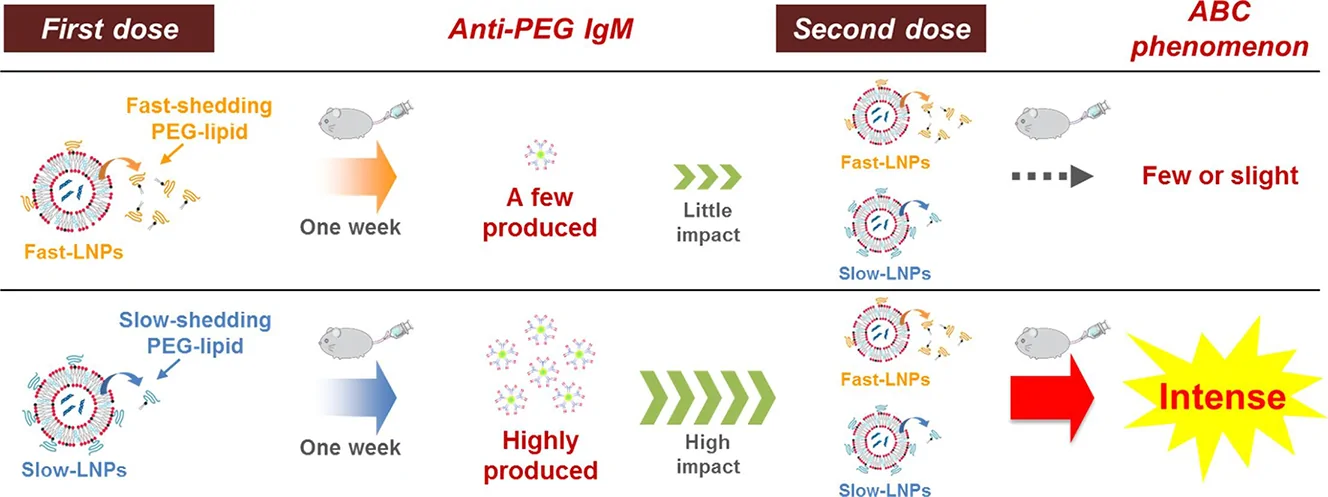
Fast-shedding PEG-lipids induce lower anti-PEG antibody production, attenuating the accelerated blood clearance phenomenon. Slow-shedding PEG-lipids conversely induce greater anti-PEG antibody production, leading to accelerated clearance upon multiple injections. Reproduced with permission [33]. Copyright 2020, Elsevier.

**FIGURE 7 |:**
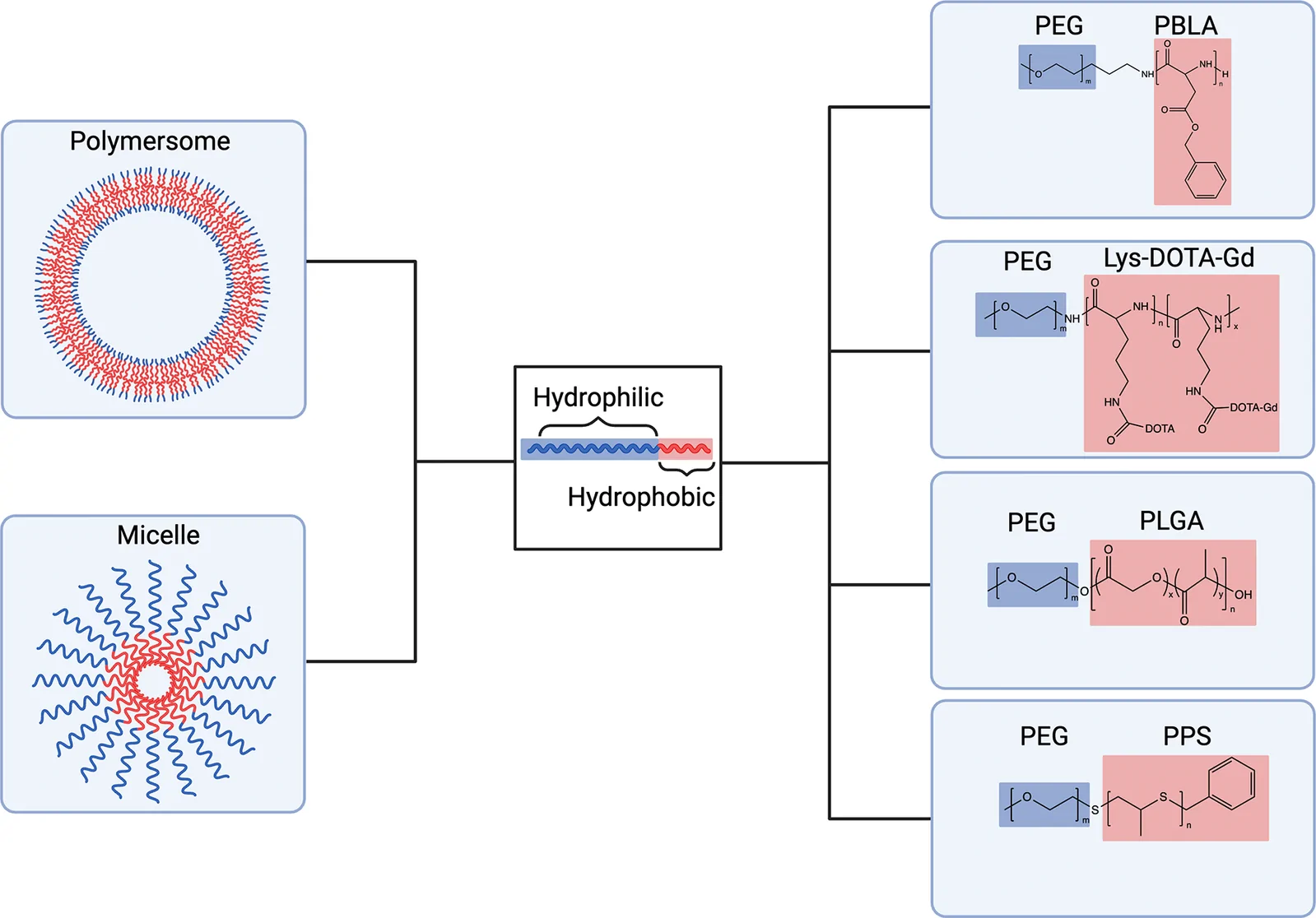
Amphiphilic block copolymers self-assemble into nanostructured carriers which may ameliorate anti-PEG immunogenicity.

**FIGURE 8 |:**
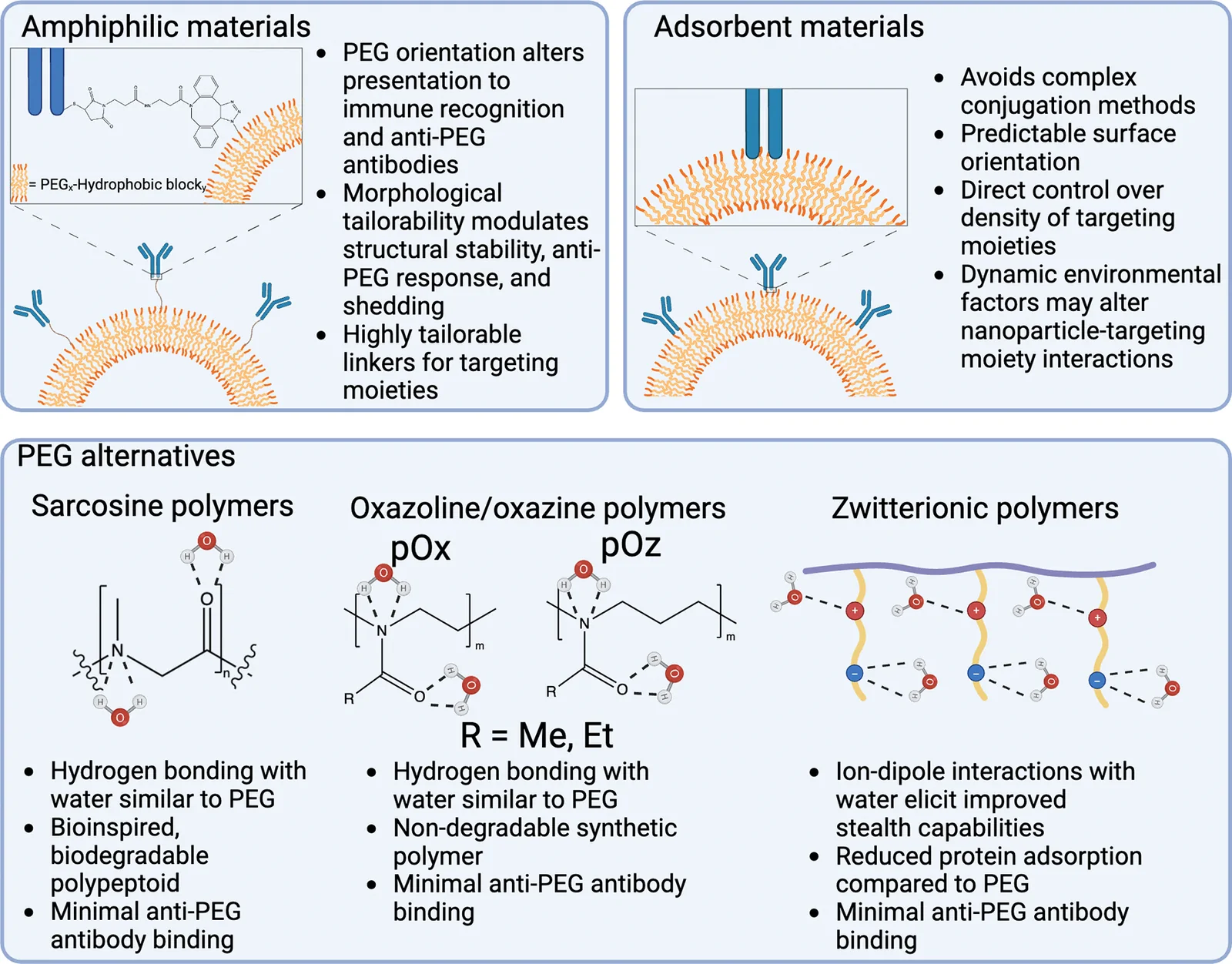
Alternative synthetic materials can avoid anti-PEG antibody recognition and alleviate accelerated blood clearance.

**FIGURE 9 |:**
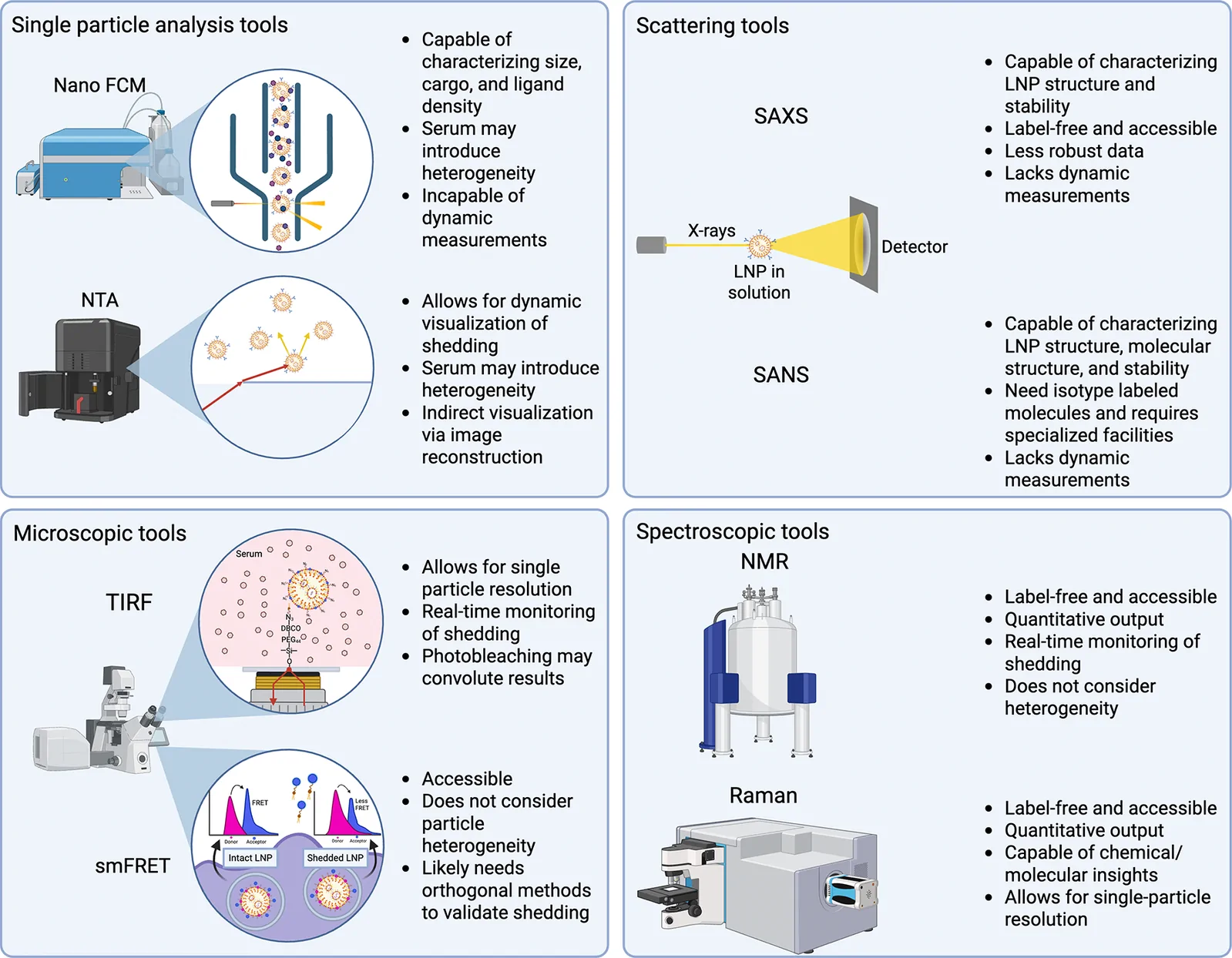
Single-particle, microscopic, scattering, and spectroscopic tools can be used to quantify shedding.

**FIGURE 10 |:**
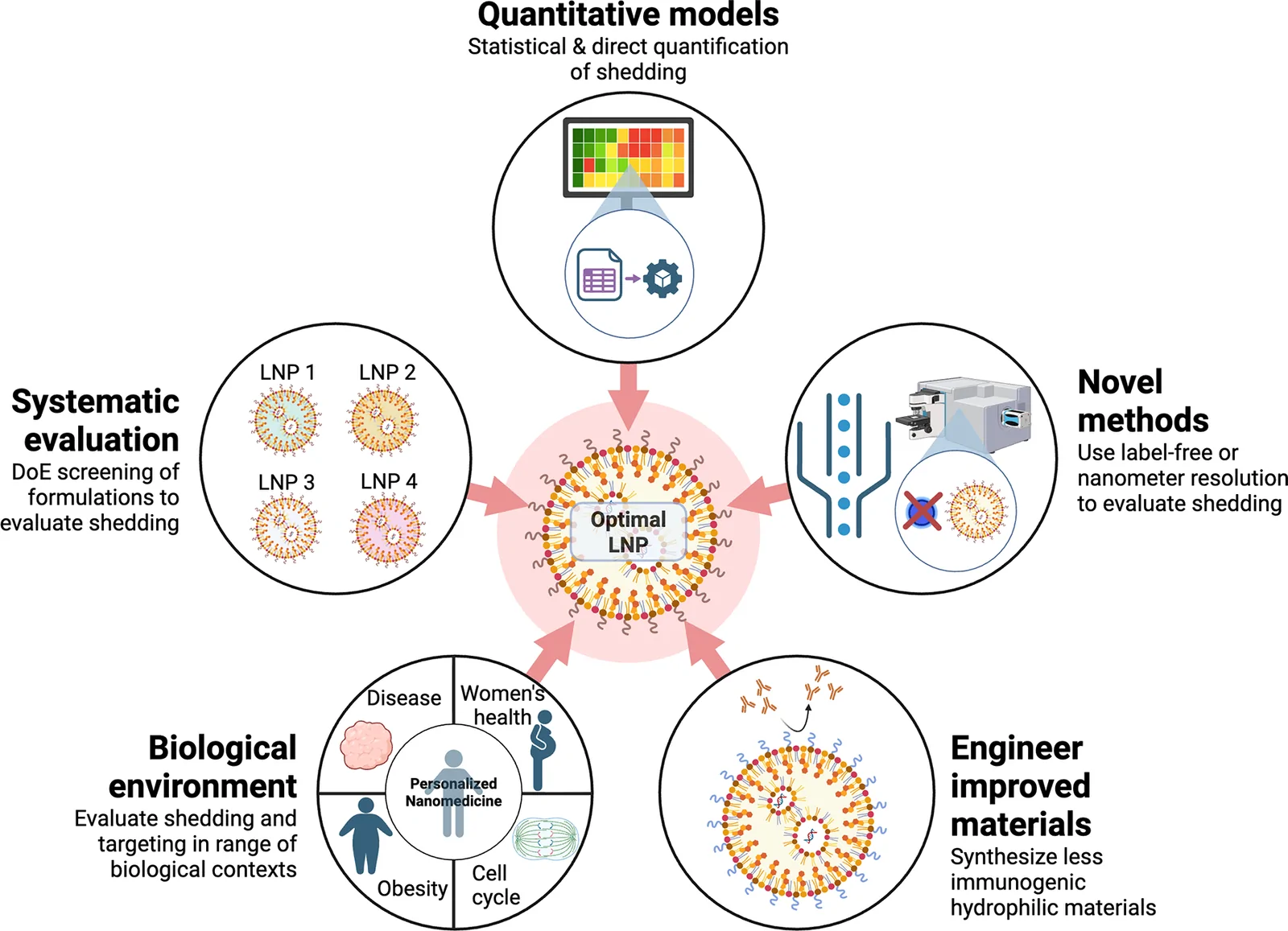
Future directions for preclinical PEG-lipid shedding studies for researchers in nanotechnology to implement.

**Table 1.** Summary of lipid chemistries found to affect targeting and shedding

Chemistry	Reference	Direct quantification of shedding (Yes/No) and methods?	Findings	Implications in shedding
Acyl length	^12^	Yes. H-NMR	LNPs containing longer acyl PEG-lipids associate most strongly; shorter acyl PEG-lipids shed to a greater extent and form micelles	• Longer acyl-chained PEG-lipids are proposed to shed less from LNPs • LNPs with long acyl PEG-lipids increase systemic exposure with elongated delivery kinetics, but reduce organ and cellular delivery • LNPs with long acyl PEG-lipids do not induce robust vaccine responses
	^31^	Yes. Radiolabeled PEG-lipids; size exclusion chromatography	Half-lives increase with longer acyl length; long acyl lengths have lower liver accumulation	
	^30^	No. *in vivo* biodistribution; protein expression	Longer acyl length increases accumulation in spleen and injured brain tissue	
	36	No. Immunofluorescence; protein expression	Half-lives increase with longer acyl length; Longer acyl lengths show better organ targeting	
	38	No. Protein expression; fluorescence microscopy	Longer acyl chains increase systemic siRNA exposure; Longer acyl chains have lower cellular uptake and siRNA delivery to liver; longer acyl chains elongate kinetics	
	39	No. Fluorescence microscopy; protein expression; *in vivo* biodistribution; cell uptake; vaccine model	Endosomal escape, transfection efficiency, and response to antigen are reduced for long acyl chains; intermediate acyl lengths show improved transfection and robust response to antigen	
	40	No. Protein expression; fluorescence microscopy	Longer acyl length results in less transfection efficiency	
Branching	41	No. Fluorescence microscopy; protein expression; *in vivo* biodistribution	Increased branching results in reduced endosomal escape and subsequent transfection; decreased branching allows for PEG-lipid to dissociate from particle and trigger endosomal escape	• Branched PEG-lipids may increase anchoring to LNP
Saturation	49	No. Computational modeling; fluorescence spectroscopy	Less saturation of PEG-lipid causes more membrane permeability; encapsulated molecules released more rapidly	• Unsaturated PEG-lipids decreases anchoring to LNP which may increase propensity to shed • Saturated and unsaturated PEG-lipids phase separate based on similar saturations
	50	No. Fluorescence recovery after photobleaching	Unsaturated PEG-lipids exhibit greater membrane diffusion compared to saturated lipids	
	59	No. Forester resonance energy transfer; cytotoxicity	Vesicles with a mix of saturated and unsaturated lipids exhibit phase separation; targeted PEG-lipids of similar saturation aggregate together	
	63	No. Protein expression; vaccine model	Intermediate degree of saturation (3 unsaturated bonds) in ionizable lipid has greatest mRNA vaccination efficacy in comparison to those with 1, 2, and 4 unsaturated bonds	
	66	No. Protein expression; Forester resonance energy transfer; *in vivo* biodistribution	Unsaturated ionizable lipid shows 18-fold increase in mRNA expression	

**Table 2.** Summary of PEG chemistries found to affect targeting and potential effects on shedding

Chemistry	Reference	Direct quantification of shedding (Yes/No) and methods?	Findings	Implications in shedding
Length	91	No. Small angle X-ray scattering; dynamic light scattering; field flow fractionation	Increasing PEG density can shield ligands from their receptors	• Increasing PEG length and density reduces protein adsorption which may also reduce shedding • Increasing PEG length enhances circulation and reduces uptake into off-target cells, but also potentially target cells • Increasing PEG length beyond a critical mass may not further reduce non-specific uptake
	83	No. Cell uptake; intravital imaging	Increased PEG density and length result in lower cell uptake in leukocytes	
	84	No. NMR; Cell uptake	Brush conformations reduce protein adsorption and non-specific uptake	
	85	No. Coarse-grained simulations	Low PEG lengths and densities enable proteins to bind to lipid bilayers	
	93	No. Small angle X-ray scattering; LC-MS/MS; Cell uptake	Protein corona is not further reduced for PEG lengths greater than 5 kDa	
Branching	94	No. Multiple particle tracking; Cell uptake	PEG branching increases diffusion of NPs through dense matrices but does not affect cell uptake	• PEG branching may lead to lower cell uptake and faster clearance PEG • PEG branching may help reduce protein adsorption to reduce shedding • PEG branching may help LNPs accumulate at tumor sites
	100	No. Cell uptake; *in vivo* pharmacokinetics; Computational molecular dynamics	LNPs with branched PEG exhibit lower cell uptake; PEG branching leads to faster clearance and reduced protein adsorption	
	101	No. Cell uptake; *in vivo* pharmacokinetics, biodistribution, and tumor model	Pharmacokinetics are comparable between branched and linear PEG; LNPs with branched PEG exhibit reduced accumulation in liver and spleen and increased accumulation in tumor	

**Table 3.** Summary of biological mechanisms in shedding

Mechanism	References	Direct quantification of shedding (Yes/No) and methods?	Findings	Implications in shedding
Protein corona	23	No. *In vivo* biodistribution; protein expression	SORT lipid recruits specific proteins to LNP surface and dictates organ tropism; PEG-lipid desorption is needed for effective protein binding to the surface	• Protein corona may act by adsorbing serum proteins to the surface to create passively targeted LNPs • Protein corona may interfere with targeting ligand-receptor interactions in both active and passive targeting contexts
	40	No. Protein expression; cell uptake; fluorescence microscopy	PEG-DMG shedding is needed for ApoE adsorption and enhanced cell uptake	
	106	No. Cell uptake	Protein corona causes loss of targeting in transferrin-functionalized NPs; Serum interrupts ligand-receptor interaction	
	107	No. Cell uptake	Actively targeted NPs are shielded by protein corona, while passively targeted NPs acquire stealth coating	
	110	No. Cell uptake; *in vivo* biodistribution and tumor model	NPs engineered to reduce protein adsorption enhance accumulation at tumor site	
Anti-PEG & ABC	117	No. Cell uptake; *in vivo* biodistribution and tumor model; ELISA	Repeated injection of >2 kDa PEG exacerbates ABC; greater anti-PEG antibody production for >1 kDa PEG	• Initial injections of longer PEG chains induce higher anti-PEG titers; subsequent injections exacerbate anti-PEG production and ABC, leading to enhanced shedding • Labile PEG-lipids may avoid ABC due to fast diffusion away from the particle once in contact with serum proteins, but may reduce targeting capabilities • Linkages between PEG and hydrophobic components are being explored, but their effects on attenuating ABC remain unclear
	118	No. Cell uptake; *in vivo* biodistribution and tumor model; ELISA	Branched PEG attenuates ABC; pharmacokinetics is prolonged; complement activation is comparable to untreated controls	
	123	No. *in vivo* pharmacokinetics; ELISA	ABC is more pronounced for liposomes with high PEG density	
	33	Yes. H-NMR; *in vivo* biodistribution; ELISA; protein expression	Fast-shedding lipids attenuate ABC in subsequent administrations of all PEGylated LNP types	
	127	No. ELISA; protein expression; *in vivo* biodistribution	Fast-shedding lipids reduce immunogenicity and maintain targeting and pharmacokinetics	
	128	No. *in vivo* pharmacokinetics	Ester linkages between PEG and cholesterol can attenuate ABC	
	70	No. *in vivo* pharmacokinetics and biodistribution; ELISA	Ester linkages reduce anti-PEG titers, but do not attenuate ABC	

## Data Availability

Data sharing not applicable to this article as no datasets were generated or analysed during the current study.
